# Assessing Catastrophic Health Expenditures Among Uninsured People Who Seek Care in US Hospital-Based Emergency Departments

**DOI:** 10.1001/jamahealthforum.2021.4359

**Published:** 2021-12-30

**Authors:** Kirstin Woody Scott, John W. Scott, Amber K. Sabbatini, Carina Chen, Angela Liu, Joseph L. Dieleman, Herbert C. Duber

**Affiliations:** 1 Department of Emergency Medicine, University of Michigan, Ann Arbor; 2 Institute for Health Metrics and Evaluation, University of Washington, Seattle; 3 Department of Surgery, University of Michigan, Ann Arbor; 4 Department of Emergency Medicine, University of Washington, Seattle; 5 Department of Health Policy and Management, Johns Hopkins Bloomberg School of Public Health, Baltimore, Maryland

## Abstract

**Importance:**

Uninsured people uniquely rely on the emergency department (ED) for care as they are less likely to have access to lower-cost alternatives. Prior work has demonstrated that most uninsured patients are at risk of catastrophic health expenditure (CHE) after being hospitalized for life-saving care. The risk of CHE for a single treat-and-release ED visit that does not result in a hospitalization among uninsured patient encounters is currently unknown.

**Objective:**

To estimate the overall national risk of CHE among uninsured patients after a single treat-and-release ED visit from 2006 through 2017, and to characterize this risk across key traits.

**Design, Setting, and Population:**

This cross-sectional study is based on a nationally representative sample of hospital-based ED visits between 2006 and 2017 in the United States (US) from the Nationwide Emergency Department Sample (NEDS). It examined outpatient ED visits among uninsured patients.

**Main Outcomes and Measures:**

Risk of CHE for ED care defined as an ED charge that exceeds 40% of one’s estimated annual post-subsistence income.

**Results:**

From 2006 to 2017, there were 41.7 million NEDS encounters that met inclusion criteria for this analysis, equating to a nationally weighted estimate of 184.6 million uninsured treat-and-release ED encounters over this period. The median ED charge for a single treat-and-release encounter grew from $842 in 2006 to $2033 by 2017. Approximately 1 in 5 uninsured patients (18% [95% CI, 18.0%-18.0%]) were at risk of CHE for a single treat-and-release ED visit over the study period. This estimated CHE risk increased from 13.6% (95% CI, 13.6%-13.6%) in 2006 to 22.6% (95% CI, 22.6%-22.7%) in 2017. Those living in the lowest income quartile faced a disproportionate share of this risk, with nearly 1 in 3 (28.5% [95% CI, 28.5%-28.6%]) facing CHE risk in 2017. In 2017, an estimated 3.2 million patient encounters nationally were at risk of CHE after a single treat-and-release ED visit.

**Conclusions and Relevance:**

This cross-sectional analysis from 2006 to 2017 of 184.6 million uninsured treat-and-release visits found that 1 in 5 uninsured patient encounters are at risk for CHE. This risk has grown over time. Future policies designed to improve access for unscheduled care must consider the unique role of the ED as the de facto safety net and ensure that uninsured patients are not at undue risk of financial harm for seeking care.

## Key Points

**Question:**

What is the risk of a single treat-and-release emergency department (ED) visit contributing to a catastrophic health expenditure (CHE; health care costs exceeding 40% of post-subsistence income) among uninsured people?

**Findings:**

In this cross-sectional study of 41.7 million ED visits from 2006 to 2017, nearly 1 in 5 (18%) uninsured treat-and-release ED patient encounters were at risk of CHE. This risk has grown over time and disproportionately burdens those with low incomes.

**Meaning:**

Policies such as broadening financial risk protection for unscheduled care may help to mitigate CHE risk among uninsured people, who have few alternatives for care outside of the ED.

## Teaser

This cross-sectional study examines the risk of annual out-of-pocket medical expenses that exceed 40% of an uninsured individual’s income resulting from a single treat-and-release emergency department visit in the US.

## Introduction

Uninsured patients are uniquely reliant on emergency departments (EDs) as a safety net, as they may not have suitable and timely alternatives when health problems arise.^1,2,3^ Yet uninsured people frequently face higher medical bills for ED care relative to insured people, who pay discounted rates for the same services.^4,5,6^ While hospitals will often reduce medical bills for uninsured patients, the onus falls to patients to negotiate this reduction, a process that can be complicated and prolonged, and can involve aggressive collections practices.^7^

National surveys show that health care costs are a persistent, major concern to the public independent of insurance status.^8,9^ However, uninsured people are disproportionately burdened by affordability concerns, with 51% of uninsured adults in 2019 reporting that they postponed care owing to medical costs.^9^ Uninsured patients are also more likely to report ED bills as the biggest source of medical cost concerns relative to the general population.^10^ These cost concerns may partially explain why uninsured people use ED services less often than their insured counterparts.^2,11^ Yet when uninsured people opt to seek care, they have fewer options outside the ED relative to those with insurance.^2^ Their vulnerability to financial hardship owing to medical bills is further compounded by the steadily rising cost of hospital and ED care,^12^ which has faced increasing scrutiny by policy makers, the media, and patients.^13,14,15,16^

Related to these cost concerns, there is a growing body of evidence in the US literature to quantify medical bills in terms of catastrophic health expenditures (CHE),^17,18,19,20^ a term used by the World Health Organization to compare out-of-pocket health care expenses with income that has been described as an objective measure of financial toxicity in health care.^21,22,23,24^ Prior studies have shown that the majority (70%-90%) of uninsured patients are at risk of CHE when emergently hospitalized for emergency conditions such as traumatic injury or acute myocardial infarction and stroke.^17,18,19^ However, the degree to which a single ED visit (that does not result in an expensive hospitalization) may contribute to financial hardship in CHE terms for uninsured people has not yet been quantified. The objectives of this study were to (1) characterize the risk of CHE (defined as annual out-of-pocket medical expenses that exceed 40% of one’s income) resulting from a single treat-and-release ED visit among uninsured encounters in the US, (2) estimate national CHE risk variation over time (2006-2017), and (3) characterize CHE risk by encounter demographics and disease categories.

## Methods

### Study Design and Data

This cross-sectional analysis used data from the 2006 to 2017 Nationwide Emergency Department Sample (NEDS), which is part of the Agency for Healthcare Quality and Research Healthcare Cost and Utilization Project (HCUP).^25^ The NEDS is an all-payer database that captures a nationally representative sample of hospital-based EDs to yield population level trends for ED visits in the US. The study’s most recent year of data (2017) contains more than 33.5 million unweighted observations to yield estimates of the approximately 144.8 million weighted ED visits that year.^25^ Encounter-level data were cross-linked to data from the US Bureau of Labor Statistics to generate income estimates.^26^ Per guidance from the Office of Research at the University of Washington, this study relied on publicly available data sources and does not qualify as human participant research and thus did not necessitate institutional review board vetting.

### Analytic Sample

To create the analytic sample, we first identified the sample of patients who had an ED disposition that NEDS describes as “treat-and-release.” This excludes all patients that were admitted to the hospital through the ED, transferred, died in the ED, or had an unknown disposition (see eTable 2 in the Supplement). We then limited the sample to only uninsured encounters, defined for this analysis as those with an expected primary payer of “self-pay.” Following prior work, we did not include the small minority of encounters (<1%) listed as “no charge” in our uninsured sample.^17^ Encounters were then excluded that were missing data for age, sex, rurality, income quartile, or NEDS charges (eFigure 2 in the Supplement). See eTable 4 in the Supplement for an analysis of encounters with missing ED charges.

### Key Variables

#### Defining Catastrophic Health Expenditure (CHE) Risk

The primary outcome variable for this study was CHE risk for a single treat-and-release ED visit among the uninsured. Similar to prior studies, we used the World Health Organization’s definition of CHE as an out-of-pocket medical expenditure that exceeds 40% of one’s post-subsistence income.^17,18,20,21,22^ Because uninsured people will be liable up to 100% of the assigned charge (as they lack an insurer to negotiate a lower rate), the ED charge provided by NEDS is assumed to be the minimum charge for that encounter’s bill as it does not include any professional fees. Emergency department charges were converted to 2017 USD$ for all years and have been modified to replace outliers with values greater than the 99th percentile to be equivalent to the 99th percentile value. In cases where the ED charge was greater than 40% of one’s simulated estimated post-subsistence income (described below), we categorized this encounter as meeting the CHE threshold.

#### Estimating Income

A limitation of all HCUP data sets is the lack of encounter-level household income, which is a key variable for calculating CHE. Instead, encounters are assigned to a community income quartile, which represents a range of household incomes based on the patient’s zip code. This study follows an established method that uses gamma distributions to generate estimated individual income for each encounter based on this zip code income quartile variable.^17,24,27,28^ Once each encounter was assigned an income based on this method, we calculated costs of food and housing across various income thresholds based on Bureau of Labor Statistics data to estimate a post-subsistence income level per encounter, similar to prior work.^17,18^ The gamma distribution parameters and subsistence spending thresholds are shown in eFigure 1, eTable 1, and eTable 2 in the Supplement.

We then compared each post-subsistence income estimate to the encounter’s listed ED charge to determine if the ED bill exceeded the CHE threshold. Yet, because the gamma distribution method of estimating income provides only 1 possible income out of an array of possible incomes for each patient living in a given income quartile, we followed prior work that uses a microsimulation model to generate this comparison 1000 times.^17,18,20,27,28^ Each patient encounter was assigned a CHE risk of either 0 (did not meet the CHE threshold) or 1 (met the CHE threshold) during a series of 1000 simulations; the probability of CHE risk for the encounter was the average across those 1000 estimates. In eTable 6 in the Supplement, we present results from a sensitivity analysis showing CHE risk when using an alternative threshold used in prior studies, which defines CHE risk as out-of-pocket health care expenses that exceed 10% of annual income.^17,23,24,29^

All ED charges and estimated income levels were adjusted for inflation prior to calculating CHE risk. All values are presented in 2017 US Dollars ($).

#### Covariates

The NEDS provides the following variables for each encounter, which were used as covariates to characterize the analytic sample and CHE risk: sex, age, principal diagnosis, zip code income quartile, urban/rural status, hospital region, and hospital teaching status. The NEDS does not report race or ethnicity, so we are unable to include these variables in our analyses.

### Statistical Analyses

We began by evaluating relevant patient demographics and hospital traits across the analytic sample of uninsured ED treat-and-release patient encounters. Trends over time are shown in eFigures 3, 4, 5, 6, 7, 8, 9, and 11 in the Supplement. Second, we quantified CHE risk for the entire cohort and then over time. Third, we estimated CHE risk for each subgroup using survey-weighted linear regression models with year fixed effects to adjust for secular trends. Fourth, we computed national estimates of the total number of uninsured treat-and-release population at risk of CHE by year across each of the 4 income quartiles. Last, to generate hypotheses related to CHE risk by condition, we completed an exploratory analysis of CHE risk by principal diagnosis for the most recent year of data (2017). To do so, we mapped each encounter’s principal diagnosis to one of the 21 *International Statistical Classification of Diseases, Tenth Revision, Clinical Modification (ICD-10-CM)* categories for that year, calculated the prevalence of the diagnosis category in the sample, and ranked each diagnosis category by CHE risk (see eTable 7, eTable 8, and eFigure 12 in the Supplement).

Encounter-level discharge survey weights were used for all analyses to generate national estimates. All 2-sided *P* values were considered statistically significant if below the 0.05 cutoff. All analyses relied on Stata version 15.0.^30^

## Results

From 2006 to 2017, there were 41.7 million NEDS encounters that met inclusion criteria for this analysis, equating to a nationally weighted estimate of 184.6 million uninsured treat-and-release ED encounters over this period (eTable 3, eFigure 2 in the Supplement). Characteristics of these uninsured treat-and-release ED encounters are shown in Table 1 and displayed over time in the Supplement (eFigures 3, 4, 5, 6, 7, 8, and 9 in the Supplement). The distribution was roughly split between male (51.1%) and female (48.9%). The majority were between the ages of 20 and 44 years (64.4%). A plurality fell within the lowest income quartile (42.4%), with relatively few coming from the highest income quartile (9.3%). When creating the analytic sample, 12.2% of available treat-and-release ED encounters were excluded owing to missing ED charges (eFigure 2 in the Supplement). We found no substantive encounter-level differences between the final analytic sample and those that had been excluded based on missing ED charge alone. For hospital traits, encounters from the West were disproportionately missing charges (eTable 4 in the Supplement).

**Table 1.** Individual and Facility Demographics of Treat-and-Release Emergency Department Uninsured Patient Encounters, 2006-2017

Demographic	Patients, %
No.	
Unweighted	41 729 750
Weighted	184 636 296
Sex	
Male	51.1
Female	48.9
Age, y	
Median	31.0
Mean	32.3
Age groups, y	
<20	14.3
20-44	64.4
45-64	20.3
≥65	1.1
Income quartile	
4 (highest)	9.3
3	18.8
2	29.5
1 (lowest)	42.4
Rurality	
Urban	46.8
Suburban	46.0
Rural	7.2
Hospital region	
Northeast	16.3
Midwest	21.9
South	57.9
West	3.9
Teaching status	
Metro	
Teaching hospital	45.6
Nonteaching hospital	36.0
Rural	18.4
ED charge, $	
Mean (SD)	2332 (3027)
Median (IQR)	1259 (632-2687)
Estimated median income for ED encounters from each income quartile, $	
4 (highest)	122 852
3	71 875
2	54 700
1 (lowest)	43 658

Abbreviation: ED, emergency department.

Authors’ interpretation of data from the Nationwide Emergency Department Sample from 2006 to 2017. The analytic sample of 41 729 750 equates to a weighted estimate of 184 636 296 uninsured treat-and-release encounters in hospital-based EDs in the United States from 2006 to 2017. All values are indexed to and presented in 2017 USD$.

In 2006, the median ED charge for an uninsured treat-and-release ED encounter was estimated at $842, whereas this had grown to $2033 by 2017 (141% relative increase over the study period). For the uninsured treat-and-release sample, the annual household income level (drawn from a single iteration of the microsimulation model used to estimate income) was estimated at $65 435 in 2006 and decreased to $59 826 by 2017 (9% relative decrease; eTable 5, eFigure 10 in the Supplement).

**Figure 1.**
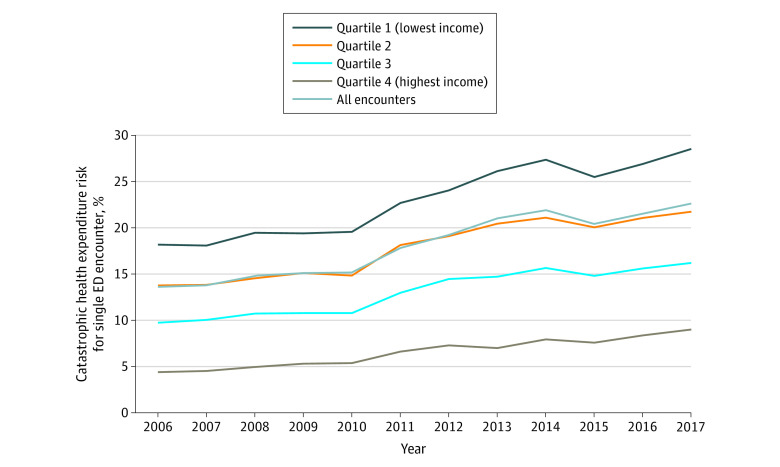
Risk of Catastrophic Health Expenditure Among Uninsured Treat-and-Release Emergency Department Encounters, by Year and Income Quartile

An estimated 18.0% (95% CI, 18.0%-18.0%) of uninsured treat-and-release ED patient encounters were at risk of receiving an ED bill that met CHE criteria for a single treat-and-release ED visit over the study period. The estimated CHE risk increased from 13.6% (95% CI, 13.6%-13.6%) in 2006 to 22.6% (95% CI, 22.6%-22.7%) in 2017, a relative increase of 66% over that period (Figure 1). When applying an alternative CHE definition that has been used in prior studies^17,23,24^ (ie, charges that exceed 10% of estimated annual income), the present study showed even higher levels of CHE risk in recent years. For instance, in 2017, 31.0% (95% CI, 31.0%-31.0%) of the uninsured treat-and-release ED population met this alternative CHE risk criteria, whereas this level was 22.6% for that same year using our primary definition of CHE risk (see eTable 6 in the Supplement).

After accounting for year variation, this study showed that the CHE risk among the uninsured treat-and-release population was relatively higher among older age groups, lower-income groups, those living outside of urban areas, those provided care at metropolitan teaching hospitals, and those in the South, as compared with their respective reference groups (Table 2; eTable 9 in the Supplement). When assessing CHE risk by community income quartile, those in the highest income quartile had the lowest overall CHE risk (6.6% [95% CI, 6.6%-6.6%, *P* < .001]) whereas those from the lowest income quartile faced the highest CHE risk (22.9% [95% CI, 22.9%-22.9%, *P* < .001]) (Table 2). Risk of CHE increased over time across all income groups. Those in the highest income quartile had a CHE risk of 4.4% (95% CI, 4.4%-4.4%) in 2006, which then grew to 9.0% (95% CI, 9.0%-9.0%) in 2017. Those in the lowest income group witnessed the greatest absolute increase in CHE risk over time (18.2% [95% CI, 18.2%-18.2%] in 2006 to 28.5% [95% CI, 28.5%-28.6%] in 2017) (Figure 1).

**Table 2.** Overall Risk of Catastrophic Health Expenditure and by Demographic and Facility Traits, Adjusted for Year

Group	CHE risk, % (95% CI)	*P* value
Sex		
Male	17.8 (17.8-17.8)	[Reference]
Female	18.2 (18.2-18.2)	<.001
Age groups, y		
<20	14.7 (14.7-14.7)	[Reference]
20-44	18.0 (18.0-18.0)	<.001
45-64	20.3 (20.3-20.3)	<.001
≥65	20.0 (20.0-20.0)	<.001
Income quartile		
4 (highest)	6.6 (6.6-6.6)	[Reference]
3	13.0 (13.0-13.0)	<.001
2	17.7 (17.7-17.7)	<.001
1 (lowest)	22.9 (22.9-22.9)	<.001
Rurality		
Urban	17.6 (17.6-17.6)	[Reference]
Suburban	18.3 (18.3-18.3)	<.001
Rural	18.2 (18.2-18.2)	<.001
Hospital region		
Northeast	15.1 (15.1-15.1)	[Reference]
Midwest	17.2 (17.1-17.2)	<.001
South	19.1 (19.1-19.1)	<.001
West	18.5 (18.5-18.5)	<.001
Teaching status		
Metro		
Teaching hospital	18.6 (18.6-18.6)	[Reference]
Non-teaching hospital	17.9 (17.9-17.9)	<.001
Rural	17.5 (17.5-17.5)	<.001

Abbreviation: CHE, catastrophic health expenditure.

Overall unadjusted CHE for all uninsured treat-and-release encounters from 2006 to 2017 was 18.0% (95% CI, 18.0-18.0). Separate linear regression models were used for each variable to produce a predicted margin for CHE risk for each subgroup. Each of these models contained a fixed effect for year to account for secular trends.

**Figure 2.**
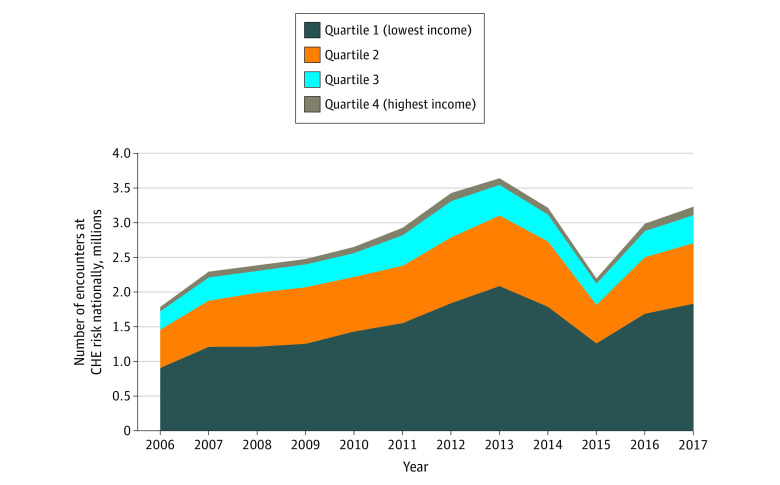
Estimated Number of Uninsured Treat-and-Release Emergency Department Encounters at Risk of Catastrophic Health Expenditure Nationally, by Year

Figure 2 summarizes the estimated number of treat-and-release ED patient encounters per year nationally who would be at risk of CHE owing to a single treat-and-release ED visit. In 2006, this equated to an estimated 1.8 million uninsured ED encounters. This grew to 3.6 million uninsured ED encounters by 2013 but subsequently dipped to 2.2 million encounters by 2015. It has since risen to 3.2 million encounters nationally as of 2017 (Figure 2).

In our exploratory analysis to assess CHE risk by disease categories using data from 2017, we found that a plurality (20.2%) of uninsured treat-and-release ED visits were injuries and/or poisonings. Among these encounters, 21.4% were at risk of CHE. See the Supplement for a list of CHE risk estimates by broad disease categories (eTables 7 and 8 in the Supplement).

## Discussion

This is the first study to our knowledge that provides national level estimates of CHE risk among uninsured patients who seek care in the ED. Between 2006 and 2017, we found that 18% of uninsured treat-and-release ED patient encounters were at risk of receiving an ED bill that met CHE criteria. This risk increased by 66% over the 11-year study period. Nationally, 3.2 million uninsured ED patient encounters were at risk of CHE after a single treat-and-release ED visit in 2017. Though CHE risk grew across all income levels, the uninsured living in the lowest income quartile shoulder the disproportionate share of this risk, with nearly a third (28.5%) of their ED visits meeting CHE criteria in 2017.

Similar to prior work that shows growth of ED charges over time,^31^ we found that median ED charges grew 141% for the uninsured treat-and-release population from 2006 to 2017. Over the same time period, estimated income levels for this group fell 9%. This increasing gap between ED charges and income levels suggests why CHE risk among the uninsured treat-and-release ED population has grown over time.

Emergency department services are generally more expensive than other sites of care given the significant resources needed to staff and maintain an ED at all times.^1,2,15,32^ Drivers of ED spending growth over time are multifactorial, ranging from increasing prices, greater intensity of services rendered in a single ED visit, and/or increasing complexity and baseline comorbidities of the presenting population.^12,30,33^ Prior work suggests that more intense and expensive workups in the ED may prevent a costly inpatient hospitalization.^1,30,34^ Given that the cost of hospitalization is nearly always greater than that of an outpatient ED visit, substituting a more intense but expedited work-up in the ED may ultimately be less financially burdensome for uninsured patients. Nonetheless, the present study findings show that uninsured people are commonly at risk of receiving a bill that can contribute to long-term economic hardship, even when a costly hospitalization is avoided. Consequently, upon receiving such a large medical bill, this may compromise the ability of the uninsured from purchasing the basic needs for their household, compromise credit scores, contribute to psychosocial stress, and potentially affect future care-seeking behavior.^35,36,37^

We caution that this analysis is not designed to assess the appropriateness of charges, actual level of payments, value of ED care, or a patient’s decision to seek ED services. Rather, it illuminates challenges in a system where individuals who lack health insurance have fewer timely or less-expensive options to access unscheduled care outside the ED.

Findings from this study can inform policy solutions designed to reduce CHE risk after seeking care in the ED, which some have described as the only federally mandated safety net in the health care system.^32^ While this study showed an increase in CHE risk over time, there was stagnation in 2015 and an overall drop in estimated numbers of uninsured treat-and-release ED encounters facing this risk nationally. This occurred in the policy context of coverage expansions under the Affordable Care Act (ACA), which reduced the number of uninsured among non-elderly adult Americans by 41% between 2010 and 2016,^38^ and has been associated with a reduction in 2 million US adults facing CHE.^39^ However, the present study suggests that this stagnation in CHE risk among treat-and-release ED visits was temporary and has risen since 2016.

This rise parallels the known uptick in the national uninsured rate since 2016,^40^ which puts more individuals at risk of lacking financial risk protection for all types of care, including the ED. A number of policies have been proposed for curtailing the remaining uninsured rate, including using a federal health exchange program for the 12 states that have not implemented Medicaid Expansion.^41,42^ However, while insurance expansion may be helpful, it is not sufficient for eliminating CHE risk. An analysis of national trends in CHE (using a data set that includes actual out-of-pocket payments) suggested that the majority of individuals in the US who experience CHE every year have private health insurance.^39^ This suggests that additional strategies are needed to mitigate CHE risk for acute, unscheduled care. Such strategies may include expanding meaningful insurance coverage for when unexpected health problems arise, bolstering state or federal incentives to curb excessive charges,^43^ mitigating aggressive financial collection practices,^44^ and further assessing payment structures for ED care to ensure that the ED is adequately compensated for its mission and service to the community while not becoming a place to which people hesitate turning to owing to costs alone.

### Limitations

The present findings should be interpreted in light of the following limitations. Notably, this study offers a conservative estimate for CHE risk among the uninsured treat-and-release ED population. For instance, the highest possible income was used to inform the income distribution within our microsimulation model within a given income quartile. Also, the ED charge within NEDS only captures facility charges. The addition of professional fees and other laboratory or radiology studies, which may not be included in the NEDS ED charge variable, would only further increase the ED charge.^14,45^ Further, this is an encounter-based (as opposed to patient-level) analysis. Since NEDS does not provide unique patient identifiers, CHE risk would only increase for individuals who seek care in the ED more than once per year (or over the study period).

Further, we could only include those encounters with an ED charge. Emergency department encounters located at hospitals in the West were disproportionately missing ED charges, thus the generalizability of these findings may not be applicable to that setting. Also, NEDS does not include race or ethnicity variables, thus we could not assess CHE risk disparities. Future work is needed to explore this further, especially in light of evidence that people of color shoulder the disproportionate burden of being uninsured^46^ as well as racial disparities in overall health spending.^47^

We limited this study to the ED treat-and-release population since prior work has demonstrated that 70% to 90% of uninsured people are at risk of CHE after being hospitalized for certain emergent conditions.^17,18^ While the goal was to identify CHE risk among those treated and released from the ED, the NEDS database does not clearly distinguish ED visits between those who are discharged directly from the ED versus those who may have a short observation stay after their ED workup but are still not hospitalized. As such, the association of short observation stays (visits that are admitted from the ED to observation units and then discharged home) with this study’s findings related to ED charges and ultimately CHE risk in our analytic sample is unknown. For ease of interpretation, we continue to use the treat-and-release language similar to prior work despite this data limitation.^48^ Also, while we completed an exploratory analysis to showcase CHE risk by broad disease categories, future research is needed to describe CHE risk by more clinically relevant disease presentations.

Finally, an important data limitation is that NEDS provides only charges, and we make no claim that the uninsured actually paid that level of allowed charges. Indeed, prior work has shown that an estimated 35% of uninsured bills are actually paid and many hospitals employ forgiveness programs for self-pay individuals.^44,49^ Nonetheless, even if not fully paid, the uninsured face many consequences when receiving a substantial medical bill (ie, psychosocial stress, being contacted by collection agencies aiming to obtain any sort of payment, compromised credit scores), making CHE risk a useful measure for this population.

## Conclusions

This cross-sectional study found that between 2006 and 2017, nearly 1 in 5 (18%) uninsured ED patient encounters were at risk of receiving a financially catastrophic bill from a single treat-and-release ED visit. In 2017, this translates to 3.2 million uninsured ED patient encounters at risk for CHE. While there are many uncertainties for the complex health care system, there will always exist a demand for unscheduled, unexpected care. If the US health care system continues to rely on the ED as the safety net for individuals who lack insurance, then policy solutions will be necessary to ensure that the system does not cause them undue financial harm.

## Notes

Supplement. eTable 1. Gini Coefficients by Year and Associated Gini-Coefficient Based Scalar eFigure 1. Gamma Distribution by Household Income by ZIP Code Community Income Quartile eTable 2. Post-Subsistence Income Calculations eTable 3. ED Encounters by Year, Unweighted Counts and Weighted National Estimates eFigure 2. Derivation of Analytic Sample eTable 4. Demographics of Uninsured Treat-and-Release ED Encounters with Missing ED charges eFigure 3. Distribution of Patient Sex Among Uninsured Treat-and-Release Encounters, by Year eFigure 4. Distribution of Age Groups Among Uninsured Treat-and-Release Encounters, by Year eFigure 5. Mean Age Among Uninsured Treat-and-Release Encounters, by Year eFigure 6. Distribution of Rurality Among Uninsured Treat-and-Release Encounters, by Year eFigure 7. Distribution of ZIP Code Income Quartile Groups Among Uninsured Treat-and-Release Encounters, by Year eFigure 8. Distribution of Hospital Region Among Uninsured Treat-and-Release Encounters, by Year eFigure 9. Distribution of Hospital Teaching Status Among Uninsured Treat-and-Release Encounters, by Year eTable 5. Comparison of ED Charges (Median and Mean) and Estimated Average of the Median Household Income Among Uninsured Treat and Release Encounters, by Year, 2017 US Dollars ($) eFigure 10. Relative Change in Median ED Charges versus Estimated Household Income, by Year eFigure 11. Distribution of Uninsured ED Treat-and-Release Encounters by Income Quartile, by Year eTable 6. Comparison of CHE Risk by Definition, Over Time eTable 7. ICD-10 CM Categories (Chapters), Code Range, and Titles eTable 8. CHE Risk by Disease Category, Ranked by Disease Prevalence in Uninsured Treat-and-Release Sample eFigure 12. CHE Risk Among Uninsured ED Treat-and-Release Encounters by Disease Category and Disease Prevalence in Sample, 2017 eTable 9. CHE Risk for Each Covariates, Unadjusted Margins Versus Margins with Year Fixed Effects
