# Supplementary material for: Assessing Catastrophic Health Expenditures Among Uninsured People Who Seek Care in US Hospital-Based Emergency Departments
Source: JAMA Health Forum. 2021 Dec 30;2(12):e214359. doi: 10.1001/jamahealthforum.2021.4359 (PMC8796980; doi:10.1001/jamahealthforum.2021.4359)
Supplement: Supplement. — eTable 1. Gini Coefficients by Year and Associated Gini-Coefficient Based Scalar eFigure 1. Gamma Distribution by Household Income by ZIP Code Community Income Quartile eTable 2. Post-Subsistence Income Calculations eTable 3. ED Encounters by Year, Unweighted Counts and Weighted National Estimates eFigure 2. Derivation of Analytic Sample eTable 4. Demographics of Uninsured Treat-and-Release ED Encounters with Missing ED charges eFigure 3. Distribution of Patient Sex Among Uninsured Treat-and-Release Encounters, by Year eFigure 4. Distribution of Age Groups Among Uninsured Treat-and-Release Encounters, by Year eFigure 5. Mean Age Among Uninsured Treat-and-Release Encounters, by Year eFigure 6. Distribution of Rurality Among Uninsured Treat-and-Release Encounters, by Year eFigure 7. Distribution of ZIP Code Income Quartile Groups Among Uninsured Treat-and-Release Encounters, by Year eFigure 8. Distribution of Hospital Region Among Uninsured Treat-and-Release Encounters, by Year eFigure 9. Distribution of Hospital Teaching Status Among Uninsured Treat-and-Release Encounters, by Year eTable 5. Comparison of ED Charges (Median and Mean) and Estimated Average of the Median Household Income Among Uninsured Treat and Release Encounters, by Year, 2017 US Dollars ($) eFigure 10. Relative Change in Median ED Charges versus Estimated Household Income, by Year eFigure 11. Distribution of Uninsured ED Treat-and-Release Encounters by Income Quartile, by Year eTable 6. Comparison of CHE Risk by Definition, Over Time eTable 7. ICD-10 CM Categories (Chapters), Code Range, and Titles eTable 8. CHE Risk by Disease Category, Ranked by Disease Prevalence in Uninsured Treat-and-Release Sample eFigure 12. CHE Risk Among Uninsured ED Treat-and-Release Encounters by Disease Category and Disease Prevalence in Sample, 2017 eTable 9. CHE Risk for Each Covariates, Unadjusted Margins Versus Margins with Year Fixed Effects [file jamahealthforum-e214359-s001.pdf]

Supplemental Online Content

Scott KW, Scott JW, Sabbatiniv AK, Chen C, Liu A, Dieleman JL, Duber HC. Assessing catastrophic health expenditures among the uninsured people who seek care in US hospital-based emergency departments. *JAMA Health Forum*. 2021;2(12):e214359. doi:10.1001/jamahealthforum.2021.4359

eTable 1. Gini Coefficients by Year and Associated Gini-Coefficient Based Scalar

eFigure 1. Gamma Distribution by Household Income by ZIP Code Community Income Quartile

eTable 2. Post-Subsistence Income Calculations

eTable 3. ED Encounters by Year, Unweighted Counts and Weighted National Estimates

eFigure 2. Derivation of Analytic Sample

eTable 4. Demographics of Uninsured Treat-and-Release ED Encounters with Missing ED charges

eFigure 3. Distribution of Patient Sex Among Uninsured Treat-and-Release Encounters, by Year

eFigure 4. Distribution of Age Groups Among Uninsured Treat-and-Release Encounters, by Year

eFigure 5. Mean Age Among Uninsured Treat-and-Release Encounters, by Year

eFigure 6. Distribution of Rurality Among Uninsured Treat-and-Release Encounters, by Year

eFigure 7. Distribution of ZIP Code Income Quartile Groups Among Uninsured Treat-and-Release Encounters, by Year

eFigure 8. Distribution of Hospital Region Among Uninsured Treat-and-Release Encounters, by Year

eFigure 9. Distribution of Hospital Teaching Status Among Uninsured Treat-and-Release Encounters, by Year

eTable 5. Comparison of ED Charges (Median and Mean) and Estimated Average of the Median Household Income Among Uninsured Treat and Release Encounters, by Year, 2017 US Dollars ($)

eFigure 10. Relative Change in Median ED Charges versus Estimated Household Income, by Year

eFigure 11. Distribution of Uninsured ED Treat-and-Release Encounters by Income Quartile, by Year

eTable 6. Comparison of CHE Risk by Definition, Over Time

eTable 7. ICD-10 CM Categories (Chapters), Code Range, and Titles

eTable 8. CHE Risk by Disease Category, Ranked by Disease Prevalence in Uninsured Treat-and-Release Sample

eFigure 12. CHE Risk Among Uninsured ED Treat-and-Release Encounters by Disease Category and Disease Prevalence in Sample, 2017

eTable 9. CHE Risk for Each Covariates, Unadjusted Margins Versus Margins with Year Fixed Effects

This supplemental material has been provided by the authors to give readers additional information about their work.

1. Conceptual Framework

The primary goal of this project was to provide national estimates of the proportion of uninsured patients who are treated and released by the ED who are at risk of a catastrophic health expenditure (CHE). The World Health Organization has defined CHE as annual out-of-pocket healthcare expenditures that exceed 40% of post-subsistence income (e.g., income that remains after accounting for food and housing costs).[1–3] We leverage prior methodologies that have quantified CHE risk, which is a component of financial toxicity, among disease-specific populations (e.g., trauma[4], cardiovascular disease[5]), to estimate this risk among uninsured ED treat-and-release patients over time.

2. Estimating Income

The methodology to estimate income in this analysis builds upon previous work that has applied estimated incomes for individuals living in income quartiles and then comparing this to listed charges. Specifically, this prior work[4,5] has utilized datasets included in the Agency for Healthcare Research and Quality Healthcare Cost Utilization Project, including the National Inpatient Sample (NIS)), which is similar to the primary data source utilized in this study: the Nationwide Emergency Department Sample (NEDS).[6] For these datasets, the only variable related to income is an estimated income for individuals living in a particular community ZIP code income quartile.

To inform the main parameters of *shape* and *scale* to estimate income in this analysis, we relied on Gini coefficients for the United States as provided by the World Bank for each year (2006-2017).[7]

Following prior work, we defined the two key parameters of shape and scale in a microsimulation model to generate income distributions for the analytic sample.[4,5,8] For shape, we used the World Bank Gini coefficients for 2006-2017 and mapped the established Gini coefficient-based scalars by year.[7,9] For scale – this was the community quartile level income estimate provided by NEDS[6] – divided by the scalar estimated for each year.

This method requires setting a mean for each ZIP code income quartile. As has been done in prior studies[4,5,10], we used the upper bound of the estimated median household income within a given ZIP code income quartile to inform the gamma distribution each year. For instance, the range of median incomes published by NEDS for the community income quartile 2 in 2017 was $44,000-$55,999.25 For every encounter who lived in ZIP code income quartile 2 in 2017, the value of $55,999 was set as the average median income level that informed the microsimulation model's shape and scale as defined above. The exception was for quartile 4 as this had no upper bound, thus this quartile's average median income level was set at the 80th percentile income level for each year, consistent with prior work.

As shown in **eTable 1**, we then mapped each Gini coefficient by year to the *shape* parameter from Shrime et al.[9] In **eFigure 1**, we display the gamma (γ) distribution by household income based on the four ZIP code community income quartiles available in the NEDS dataset (**eFigure 1**).

© 2022 Scott et al. *JAMA Health Forum.*

eTable 1. Gini Coefficients by Year and Associated Gini-Coefficient Based Scalar

Year	Gini Coefficient	Shape (Gini-Based Coefficient Scalar)
**2006**	0.414	1.585
**2007**	0.408	1.653
**2008**	0.408	1.653
**2009**	0.406	1.652
**2010**	0.400	1.744
**2011**	0.409	1.631
**2012**	0.409	1.631
**2013**	0.407	1.648
**2014**	0.415	1.568
**2015**	0.412	1.610
**2016**	0.411	1.642
**2017**	0.412	1.610

Note: Source for Gini coefficient by year: https://data.worldbank.org/indicator/SI.POV.GINI?locations=US. Source for the "shape" parameter (i.e., Gini-coefficient based scalar) comes from Shrime et al. 2016 (Table S1).

© 2022 Scott et al. *JAMA Health Forum.*

eFigure 1. Gamma Distribution by Household Income by ZIP Code Community Income Quartile

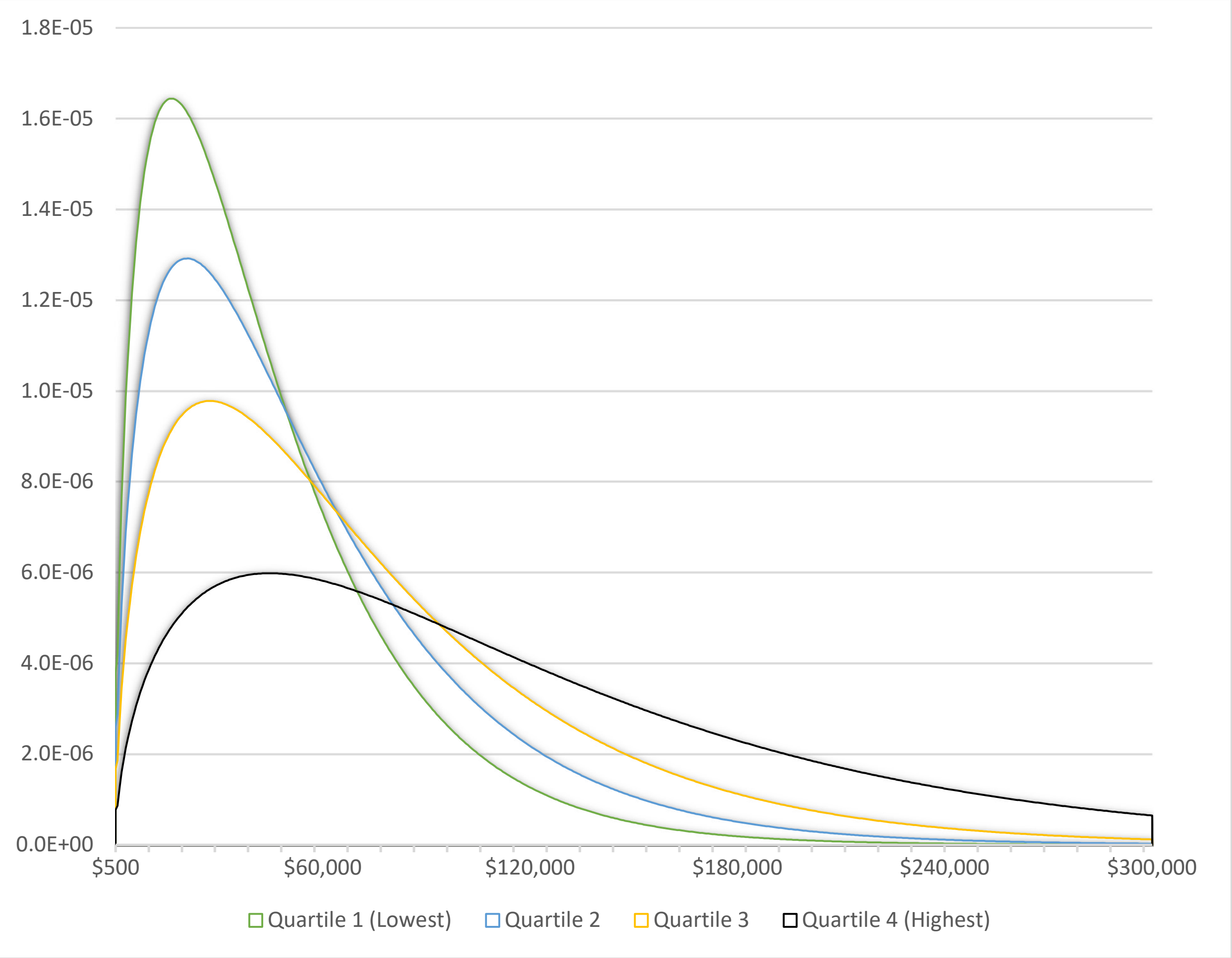

Once each encounter was assigned an income in the microsimulation model, we then estimated a post-subsistence income level per encounter, as has been done in prior work.[3] Post-subsistence income was calculated as the remaining income after taking into account costs of food and housing. Estimates for food and housing costs across various income thresholds are available from the Bureau of Labor Statistics.[9] For instance, if an encounter's estimated income was <$15,000, then 91.5% of their income was estimated to have been spent on food and housing, and the remaining balance would be defined as their post-subsistence income. As another example, if an encounter's estimated income was between $70,000-$99,999, then 37.0% of their income was estimated to have been spent on food and housing.

© 2022 Scott et al. *JAMA Health Forum.*

eTable 2. Post-Subsistence Income Calculations

Upper Limit of Income (y)	Food Costs (x1)	Housing Costs (x2)	Proportion Devoted to Food/Housing ((x1+x2)/y)
15000	3,938	9783	0.915
30000	4,806	12,817	0.587
40000	5,878	14,585	0.512
50000	6,598	16,216	0.456
70000	7,443	18,446	0.370
100000	8,385	21,933	0.303
150000	10,918	26,684	0.251
200000	12,171	35,589	0.239
376,587	16,309	47,410	0.169

3. Data

The key dataset used for this analysis comes from the publicly NEDS, which represents the largest all-payer sample of hospital-based emergency departments (ED) in the United States.[6] The NEDS databased started in 2006 and the latest year of data available when this analysis was completed was 2017. The NEDS provides a discharge weight (variable = discwt) that allows for one to use a survey-weighted analysis to produce national estimates. A summary of ED encounters by year is provided in **eTable 3**.

© 2022 Scott et al. *JAMA Health Forum.*

eTable 3. ED Encounters by Year, Unweighted Counts and Weighted National Estimates

Year	Total NEDS Encounters	Admitted to Hospital from ED	Treat-and-Release ED Encounters	Uninsured Among Treat & Release ED	Uninsured Treat & Release ED Encounters	Uninsured Treat & Release ED encounters meeting inclusion criteria	Uninsured Treat & Release ED encounters meeting inclusion criteria
	(unweighted n)	*(Weighted %)*	*(Weighted %)*	*(Weighted %)*	*(unweighted n)*	*(unweighted n)*	*(weighted n)*
**2006**	25,702,597	15.5	79.3	18.0	3,716,391	2,933,409	13,095,836
**2007**	26,627,923	15.6	81.4	19.8	4,352,447	3,673,033	16,616,558
**2008**	28,447,148	15.5	82.3	18.5	4,359,705	3,699,156	16,108,608
**2009**	28,861,047	15.2	82.7	18.0	4,367,019	3,709,326	16,345,358
**2010**	28,584,301	15.3	82.7	19.0	4,580,959	3,947,097	17,456,789
**2011**	28,788,399	15.0	83.2	17.6	4,277,603	3,631,193	16,418,259
**2012**	31,091,020	14.2	84.0	18.5	4,890,006	4,180,566	17,814,965
**2013**	29,581,718	14.3	83.9	17.8	4,514,846	3,861,309	17,317,034
**2014**	31,026,417	14.1	84.1	14.6	3,805,493	3,258,885	14,668,721
**2015**	30,542,691	13.6	77.0	11.3	2,670,217	2,281,297	10,737,323
**2016**	32,680,232	13.1	85.0	12.5	3,507,051	3,132,651	13,798,510
**2017**	33,506,645	14.0	84.1	12.8	3,729,059	3,421,828	14,258,335
**All Years**	**355,440,138**	**14.6**	**82.5**	**16.4**	**48,770,796**	**41,729,750**	**184,636,296**

Note: The unweighted count of 355,440,138 ED encounters from the Nationwide Emergency Department Sample (NEDS) estimates to a survey weighted count of 1,596,418,071 ED encounters between 2006-2017. Survey weight=discwt as provided by NEDS to obtain national estimates.

A disposition variable is provided by NEDS (variable: EDEVENT) that categorizes patients as “treat-and-release”, “admitted”, and some additional categories. We included only treat-and-release encounters (EDEVENT=1), which is the majority of ED visits. Among this subgroup, we identified those patients that were uninsured. For this analysis and following prior work that focuses on CHE in the uninsured[4], we defined the uninsured using the expected primary payer variable (PAY1) if they were “self-pay”. Of note, some studies define the uninsured population in NEDS as encounters with the expected primary payer of both “self pay” and those listed as “no charge”. However, the “no charge” group is relatively small (0.7% of the entire sample) and a heterogeneous group as these could include patients with insurance but not expected to have a charge (e.g. Medicaid patients). Since our objective was to assess CHE risk among those who were expected to not have any insurance protection among those who received a bill, this is why we included only “self-pay” in our definition of the uninsured as other studies have done.[4] Among this treat-and-release subgroup, a total of 16.4% were uninsured. Of the 48,770,796 uninsured ED treat-and-release encounters, 41,729,750 met inclusion criteria for the analysis. Further detail on how the analytic sample was derived is available in **eFigure 2**.

As noted in the **eFigure 2**, only a small number of observations were missing key variables in this analysis thus relatively few encounters were dropped due to missing data. However, the majority of dropped observations stemmed from a core variable needed for this analysis – ED charge. We provide a summary in **eTable 4** of how the population demographics vary by the full analytic sample as compared to what the sample would have looked like had these observations not been dropped. As shown in the manuscript for our primary analytic sample and again in **eTable 4**, relatively few uninsured treat-and-release encounters were in the West as compared to other regions. This representation was further limited in that the majority of uninsured treat-and-release encounters with missing ED charges also came from the West, which NEDS has documented as a feature of these data.[6]

© 2022 Scott et al. *JAMA Health Forum.*

eFigure 2. Derivation of Analytic Sample

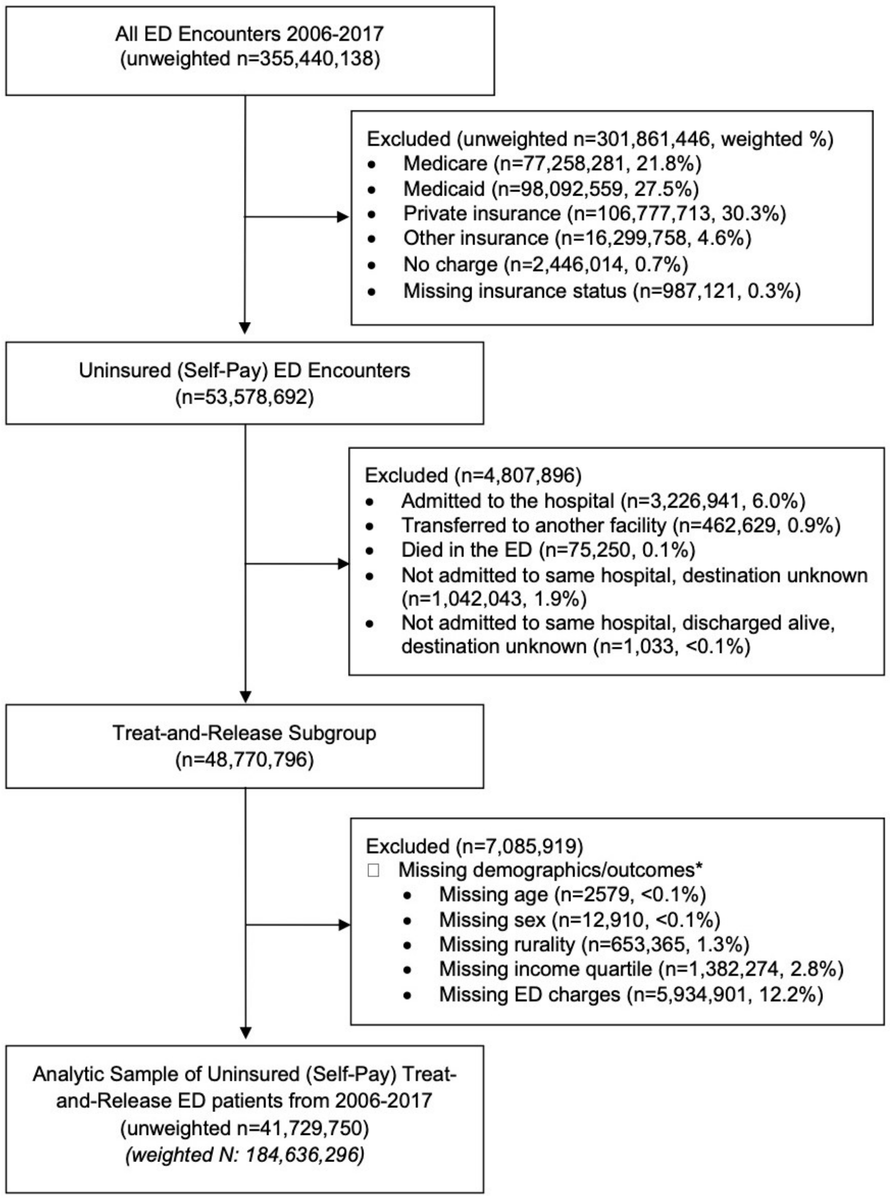

Note: *Not all values add to 100% as some observations had more than one missing value for these variables. The counts next to each variable are the raw number of observations and the percentage is the weighted percentage of the sample. See Supplement **eTable 4** for an analysis of the sample with only missing ED charges.

eTable 4. Demographics of Uninsured Treat-and-Release ED Encounters with Missing ED charges

**Demographic**		**Analytic Sample**	**Full Sample**	**Missing Only ED Charges**
		n=41,729,750	*n=47,372,968*	*n= 5,643,218*
Sex (%)	*Male*	51.1	51.4	53.3
	Female	48.9	48.6	46.7
Age (yrs)	*Median (Mean)*	31 (32.3)	31 (32.23)	30 (31.4)
Age Groups (%)	*<20 years*	14.3	15.0	19.9
	20-44	64.4	63.6	57.6
	45-64	20.3	20.3	20.7
	65+	1.1	1.2	1.8
Income Quartile (%)	*Lowest*	42.4	40.4	25.6
	Second	29.5	29.1	26.1
	Third	18.8	19.9	27.8
	Highest	9.3	10.7	20.5
Rurality (%)	*Urban*	46.8	48.5	61.0
	Suburban	46.0	44.7	35.6
	Rural	7.2	6.7	3.4
Hospital Region (%)	*Northeast*	16.3	14.5	0.7
	Midwest	21.9	19.5	1.7
	South	57.9	52.3	10.6
	West	3.9	13.8	87.0
Teaching Status (%)	*Teaching Hospital (Metro)*	43.8	43.2	38.7
	Non-teaching Hospital (Metro)	34.6	36.3	48.5
	Rural	17.6	16.5	8.1
	*Missing**	3.9	4.0	4.8

Note: Data Source: NEDS, Years: 2006-2017. The analytic sample is all uninsured treat-and-release ED encounters that have no encounter-level missing values. This is compared to the treat-and-release encounters that were excluded based on missing ED charges. Estimated income was not calculated for encounters outside of the analytic sample.

As shown in **eTable 4**, a total of 5,934,901 of treat and release encounters were missing ED charges. Once excluding those encounters with other missing values (n= 1,397,828 encounters that were missing values for age OR sex OR urban/rural designation OR ZIP code income quartile), this left a total of n=5,643,218 encounters that were missing only ED charges. The demographics of this sample that was excluded is shown in **eTable 4**. We also compare this to the full sample, had these observations not been excluded. Of note, we did not exclude the relatively

© 2022 Scott et al. *JAMA Health Forum.*

few encounters that were missing values for hospital level variables of interest (e.g., hospital teaching status) given that this was an encounter-level analysis. Since there were no missing values for hospital region among this study's analytic sample, no observations were dropped due to missing data for this variable. For teaching status, however, in 2006, approximately 55% of the analytic sample was missing hospital teaching status. However, hospital teaching status had no missing values in the remaining years (2007-2017). Given this anomaly, we did not drop any of these encounters (which otherwise had all of the remaining encounter-level variables of interest, including ED charges) based solely on missing hospital teaching status. We display the distribution of teaching status over time among the analytic sample by teaching status after excluding those missing values (see **eFigure 9** below), but these 1.9 million observations were not dropped from any remaining analyses.

4. Changes in Uninsured ED Treat-and-Release Population Over Time

The following figures **(eFigures 3-9)** illustrate how the uninsured population in the analytic sample (n=41,729,750) has changed over the study period (2006-2017). In general, the distribution appeared relatively constant over time by patient sex. For age groups, there was a decline in encounters consisting of the <20 year age group over time, possibly related to health reform insurance expansion efforts targeted at younger age groups (e.g., the dependent coverage provision of the Affordable Care Act[11]). In terms of ZIP code. This was only reflected by a slight uptick in the average age for ED uninsured treat-and release encounters over time (30.6 years in 2006 versus 33.9 years in 2017). For ZIP code income quartile, a greater proportion of uninsured treat-and-release encounters consisted of individuals living in the lowest income quartile (Quartile 1) over time (38.0% in 2006 versus 45.0% in 2006). For rurality, this was relatively constant over time, though there was a slight uptick in encounters located in urban areas in 2017. Though this study was focused on an individual-level variable (encounters), we also assessed where these encounters were located at based on some of the available NEDS hospital-level variables (region and teaching status). For hospital region, the largest share of uninsured treat-and release encounters was located in the South, and this grew over time. For hospital teaching status, a greater proportion of uninsured treat-and release encounters appeared to be from EDs affiliated with metropolitan teaching hospitals as compared to non-teaching hospitals over time.

eFigure 3. Distribution of Patient Sex Among Uninsured Treat-and-Release Encounters, by Year

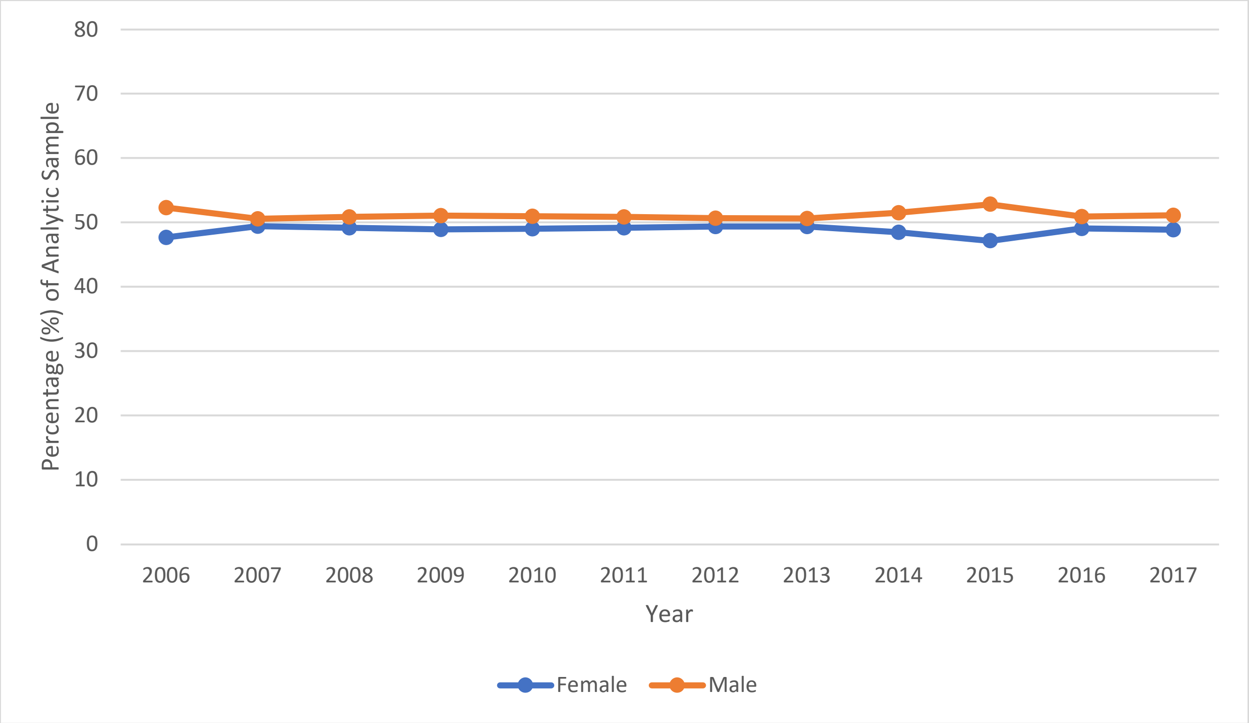

© 2022 Scott et al. *JAMA Health Forum.*

Note: Overall distribution of sex across all years in analytic sample (n=41,729,750): female (48.9%) versus male (51.1%).

eFigure 4. Distribution of Age Groups Among Uninsured Treat-and-Release Encounters, by Year

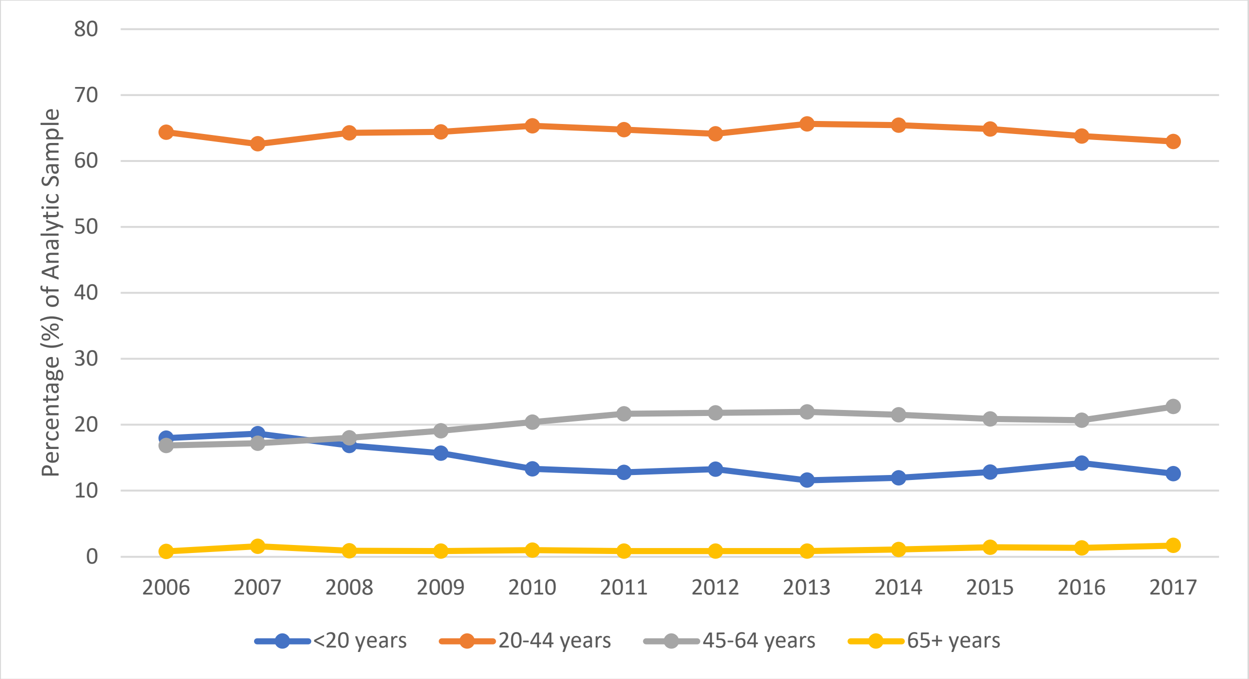

Note: Overall distribution of age groups across all years in analytic sample (n=41,729,750): <20 years (14.3%), 20-44 years (64.4%), 45-64 years (20.3%), and 65 years and above (1.1%).

eFigure 5. Mean Age Among Uninsured Treat-and-Release Encounters, by Year

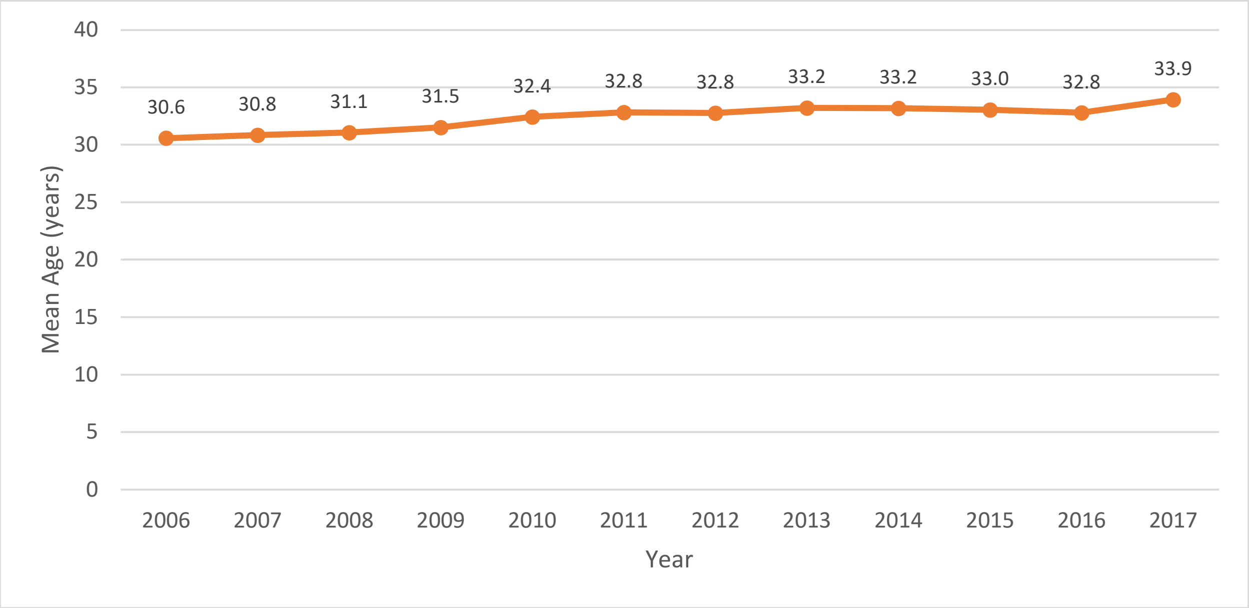

© 2022 Scott et al. *JAMA Health Forum.*

eFigure 6. Distribution of Rurality Among Uninsured Treat-and-Release Encounters, by Year

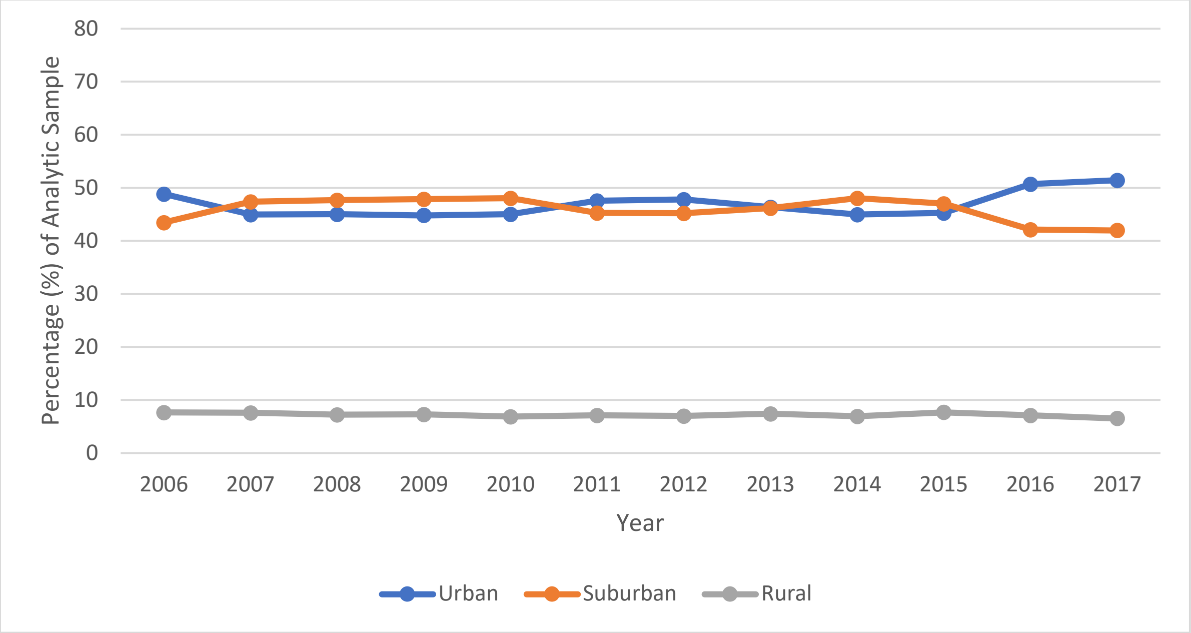

Note: Overall distribution of urban-rural designation across all years in analytic sample (n=41,729,750): urban (46.8%), suburban (46.0%), and rural (7.2%).

eFigure 7. Distribution of ZIP Code Income Quartile Groups Among Uninsured Treat-and-Release Encounters, by Year

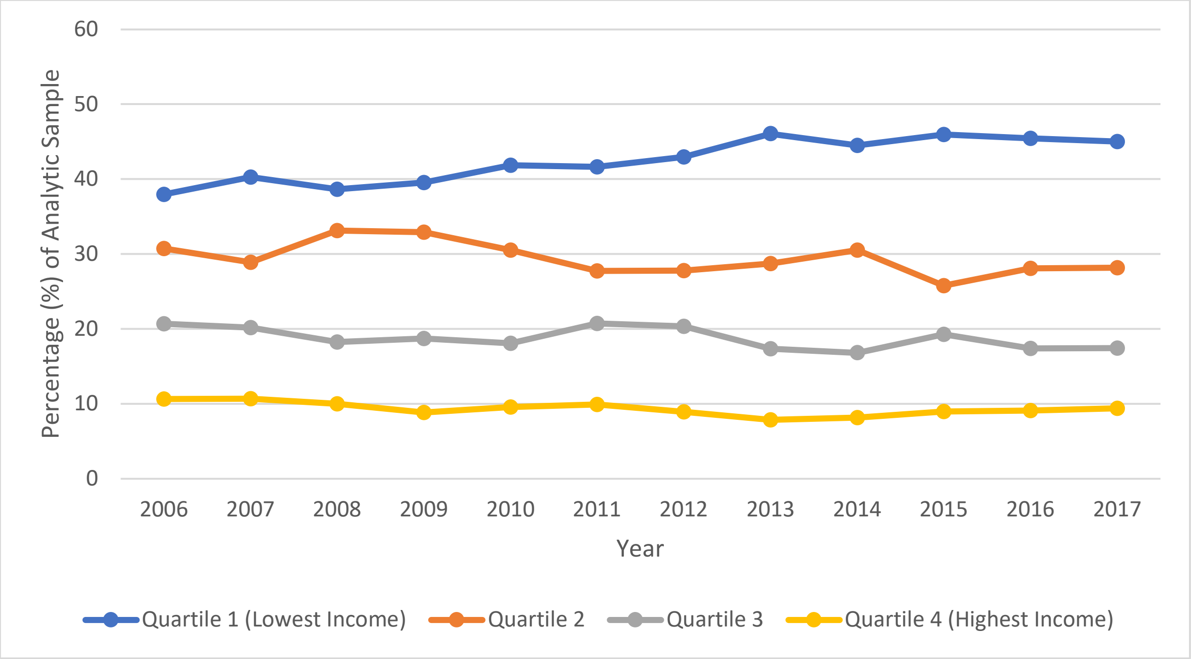

Note: Overall distribution of ZIP code income quartiles across all years in analytic sample (n=41,729,750): Quartile 1 (lowest income quartile) (42.4%), Quartile 2 (29.5%), Quartile 3 (18.8%), Quartile 4 (highest income quartile) (9.3%).

© 2022 Scott et al. *JAMA Health Forum.*

eFigure 8. Distribution of Hospital Region Among Uninsured Treat-and-Release Encounters, by Year

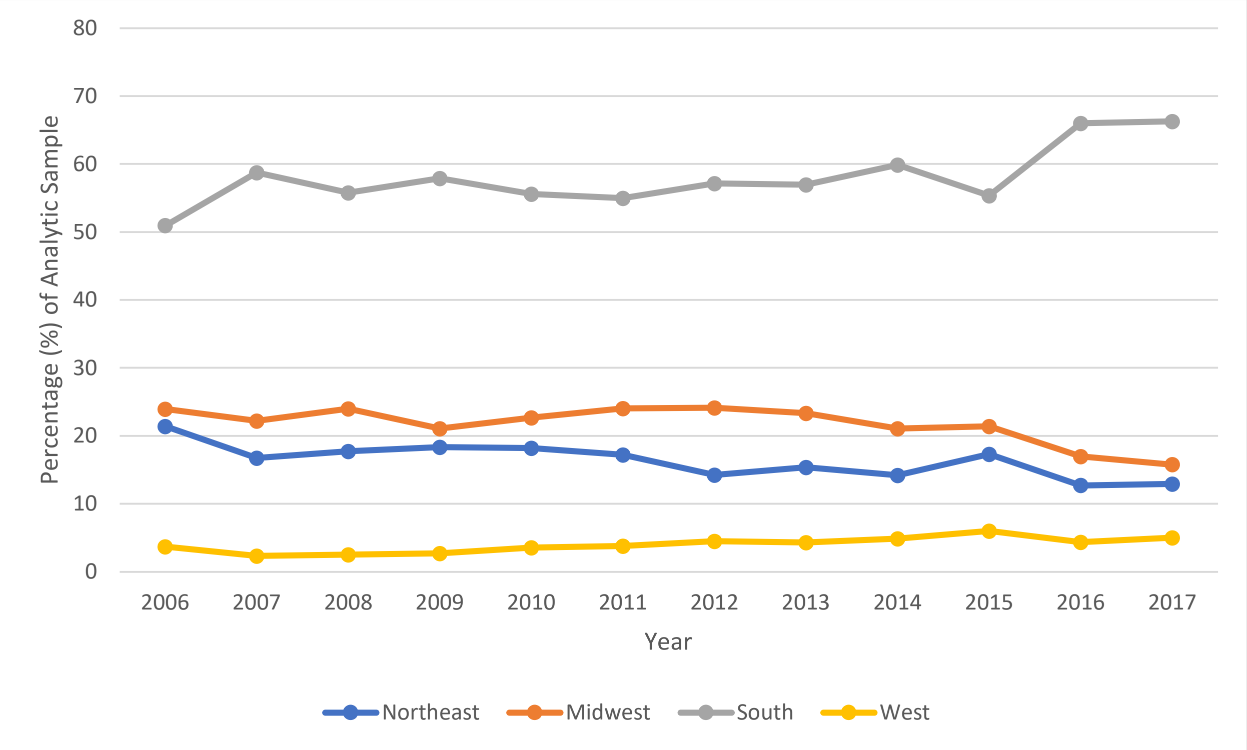

Note: Overall distribution of hospital region across all years in analytic sample (n=41,729,750): Northeast (16.3%), Midwest (21.9%), South (57.9%), and West (3.9%). As discussed elsewhere in the Supplement, the majority of encounters with missing charges (which were dropped from the analytic sample) are concentrated in hospitals located in the West. However, had these observations not been excluded, only a minority of uninsured treat-and-release encounters would have been in the West relative to other regions, primarily the South.

© 2022 Scott et al. *JAMA Health Forum.*

eFigure 9. Distribution of Hospital Teaching Status Among Uninsured Treat-and-Release Encounters, by Year

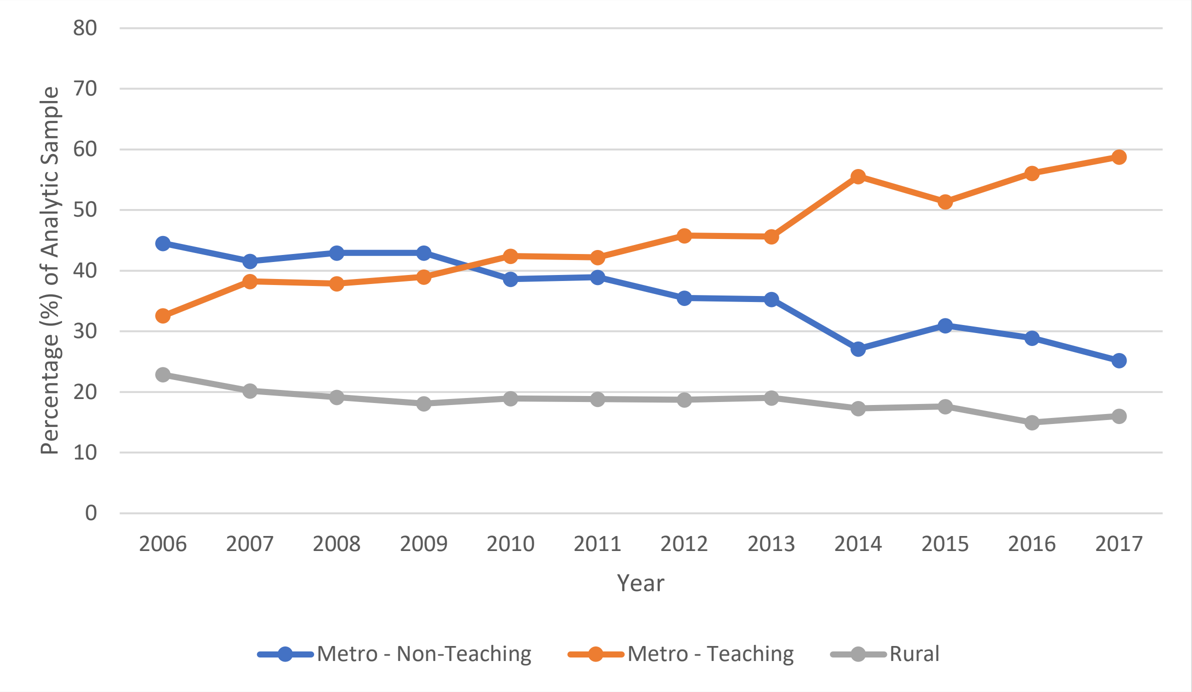

Note: Overall distribution of hospital teaching status across all years in analytic sample (n=41,729,750): Metropolitan (Metro) – Non-Teaching (36.0%), Metro-Teaching (45.6%), and Rural (18.4%). This study did not exclude any encounters from the analytic sample if they had a missing value for teaching status, which only occurred in 2006. For 2006, approximately 55% of the encounters (n=1.6 million) in the analytic sample for that year (n=2.9 million) had a missing value for teaching status, even though every other variable of interest in this study (including ED charge) was not missing. Otherwise, there were no missing values for teaching status among the analytic sample in years 2007-2017 and thus no observations from the analytic sample were excluded for what appeared to be an anomaly in 2006 for this variable. For the sake of clarity, however, the above distribution was made by only showcasing the distribution in 2006 when excluding the missing values for teaching status that year. When observing trends from 2007 onward (where there were no missing values for teaching status among the analytic sample), there appears to be a relative growth in uninsured treat-and-release encounters stemming from Eds affiliated with metro-teaching hospitals.

5. Comparison of ED Charges and Income Over Time

All values were adjusted for inflation and displayed in 2017 US Dollar terms. Any encounters that were missing ED charges (variable = TOTCHG_ED) were dropped from the analysis. To eliminate erroneous outliers in ED charges, we computed a modified ED charge that replaced those charges exceeding the 99th percentile of charges with the dollar amount of the ED charge listed at the 99th percentile.

In **eTable 5** and **eFigure 10**, we show that, even after accounting for inflation, ED charges have risen over time while the estimated median household income (one estimate was randomly drawn among the 1000 simulations to illustrate a representative example of income distributions for the analytic sample) slightly decreased. Given the makeup of the outpatient uninsured ED population in terms of ZIP code community income quartile, this relatively stable to decreasing incomes is not unexpected since fewer members living in the highest income quartile make up the analytic sample over time (**eFigure 7**, **eFigure 11**).

eTable 5. Comparison of ED Charges (Median and Mean) and Estimated Average of the Median Household Income Among Uninsured Treat and Release Encounters, by Year, 2017 US Dollars ($)

Year	ED Charges (Median) ($)	ED Charges (Mean) ($)	Estimated Income ($)
**2006**	842	1,555	65435
**2007**	908	1,686	64409
**2008**	942	1,746	61454
**2009**	1,037	1,935	62124
**2010**	1,188	2,139	62073
**2011**	1,203	2,166	58207
**2012**	1,288	2,330	56023
**2013**	1,418	2,519	53888
**2014**	1,539	2,757	55537
**2015**	1,549	2,736	57791
**2016**	1,833	3,249	58892
**2017**	2,033	3,496	59826

Note: All charges and income values are presented in 2017 US Dollars ($). The estimated income is the average of the estimated median household income after applying a microsimulation model to estimate income for each encounter based on ZIP code income quartile levels. The estimated income data shown above comes from one randomly selected draw from the 1,000 simulations, which allowed us to provide discrete estimates by year.

eFigure 10. Relative Change in Median ED Charges versus Estimated Household Income, by Year

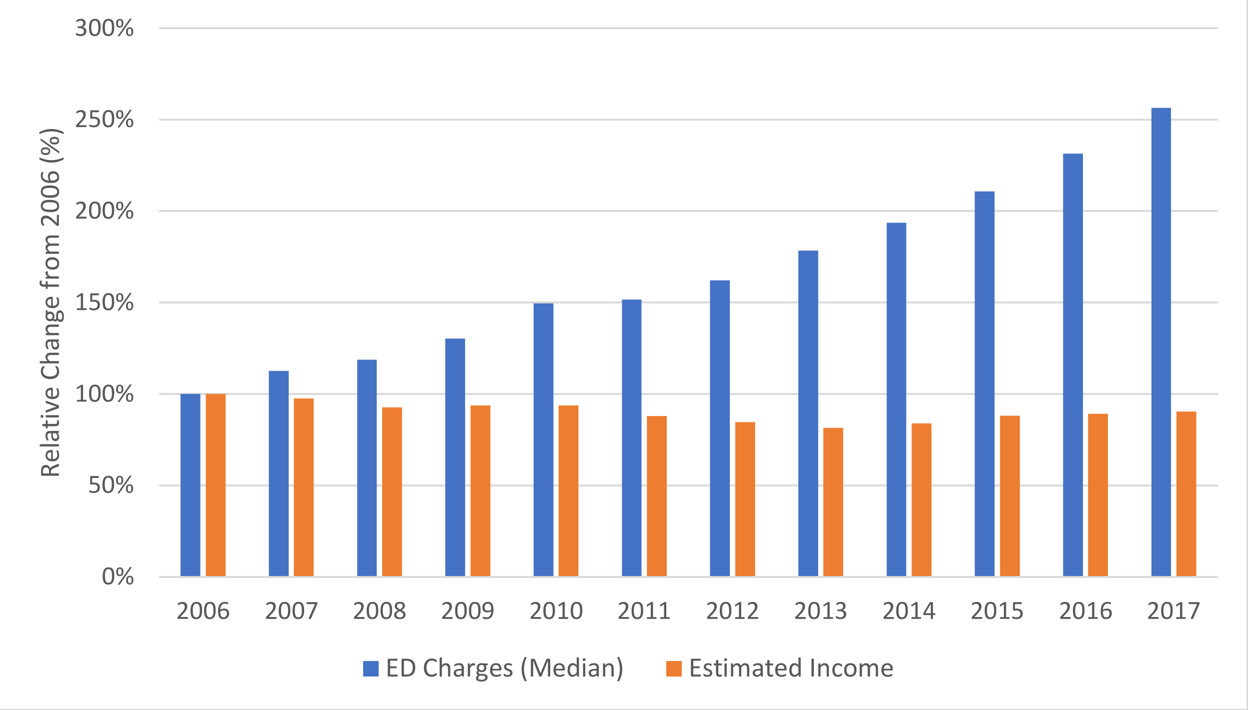

Note: All values are in 2017 US Dollars and have been adjusted for inflation. This shows the relative change from the baseline year of 2006 for both median ED charges and estimated household income in the analytic sample, by year. Compared to 2006, median ED changes increased by 141% by 2017 whereas estimated household income decreased by 9% between 2006 and 2017.

© 2022 Scott et al. *JAMA Health Forum.*

eFigure 11. Distribution of Uninsured ED Treat-and-Release Encounters by Income Quartile, by Year

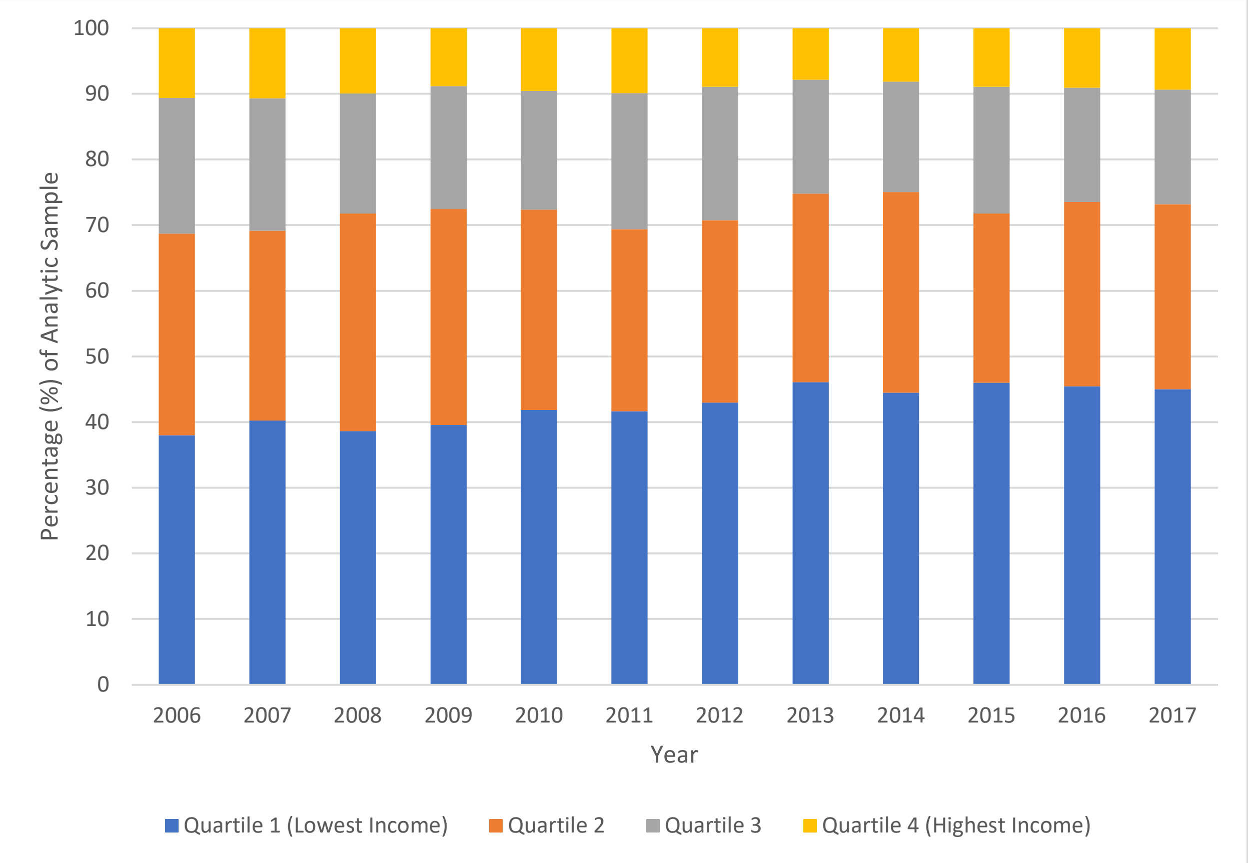

Note: This figure is another depiction of the analytic sample over time (stacked bar chart format), which we illustrate in a line graph in **eFigure 7**. It demonstrates that a slightly greater proportion of the analytic sample consists of encounters from lower-income quartiles over time.

6. CHE Risk by Definition

For the purposes of this study, a Catastrophic Health Expenditure (CHE) is calculated by comparing the listed ED charge for each encounter and comparing this to the estimated income for each encounter (which was calculated from the microsimulation model based on each encounter's ZIP code income quartile, as described in detail above). Though there remains a number of ways in how CHE has been defined and operationalized in prior studies, we employ the World Health Organization's (WHO) definition of CHE which has been described as out-of-pocket spending on healthcare that exceeds 40% of one's post-subsistence income (e.g., income that has accounted for estimated costs of housing and food).[2,3] For this study, we define "CHE 40 Risk" as an ED charge for a single encounter that exceeds 40% of the encounter's estimated annual post-subsistence income.

An alternative definition that has been used in CHE/financial hardship literature is out-of-pocket healthcare spending that exceeds 10% of annual overall income.[4,12–14] For this alternative definition, we defined what we call "CHE 10 Risk" as the proportion of encounters that had an ED charge that exceeded 10% of their estimated annual household income.

For this study, CHE 40 Risk is at a similar level to that of CHE 10 Risk in 2006 (13.6% and 13.4%, respectively), but CHE 10 Risk grows to an absolute higher level over time as compared to CHE 40 Risk. Specifically, 31.0% of the analytic sample met criteria for CHE 10 Risk in 2017 as compared to only 22.6% of the sample meeting CHE 40 Risk that same year (**eTable 6**).

© 2022 Scott et al. *JAMA Health Forum.*

eTable 6. Comparison of CHE Risk by Definition, Over Time

	**CHE 40 Risk**		**CHE 10 Risk**	
**Year**	Mean (%)	95% CI	Mean (%)	95% CI
**2006**	13.6	(13.6-13.6)	13.4	(13.4-13.4)
**2007**	13.8	(13.8-13.8)	14.1	(14.1-14.2)
**2008**	14.8	(14.8-14.8)	15.3	(15.3-15.3)
**2009**	15.1	(15.1-15.1)	16.5	(16.5-16.5)
**2010**	15.2	(15.2-15.2)	17.9	(17.9-17.9)
**2011**	17.8	(17.8-17.8)	20.2	(20.2-20.2)
**2012**	19.2	(19.2-19.2)	22.2	(22.2-22.2)
**2013**	21.0	(21.0-21.0)	24.9	(24.9-24.9)
**2014**	21.9	(21.9-21.9)	26.8	(26.8-26.8)
**2015**	20.4	(20.4-20.5)	25.5	(25.5-25.6)
**2016**	21.6	(21.6-21.6)	28.9	(28.9-28.9)
**2017**	22.6	(22.6-22.7)	31.0	(31.0-31.1)
All Years	***18.0***	***(18.0-18.0)***	***21.2***	***(21.1-21.2)***

Note: CHE=Catastrophic Health Expenditure (CHE). CHE 40 Risk = ED charge that exceeds 40% of estimated annual post-subsistence household income (i.e., income that remains after accounting for food and housing costs). CHE 10 Risk = ED charge the exceeds 10% of estimated annual household income.

7. CHE Risk by Diagnosis Category

The primary focus of this project was to showcase CHE risk nationally and over time. However, we also conducted an exploratory analysis to see if and how CHE risk may vary by diagnosis categories. We limited this analysis to our most recent year of data (2017). We mapped each encounter to one of the twenty-one categories (chapters) of ICD-10 CM codes as defined in 2017 by the primary diagnosis (variable dx1).[15] Of the 3,421,828 observations from our analytic sample in 2017, only 1783 were missing a primary diagnosis code and an additional 32 had an invalid value for the listed code. This equates to a total of 3,420,013 observations (99.9%) were matched to one of the twenty-one ICD10-CM categories.

In **eTable 7**, we present the risk of CHE based on both the primary definition used in this analysis (CHE 40 Risk) as well as an alternative definition (CHE 10 Risk), as defined above. We display these results sorted by the prevalence of the sample that was mapped to a particular ICD-10 category. For instance, 20.2% of the 2017 uninsured treat-and-release encounters in our analytic sample had a primary diagnosis code that mapped to the ICD-10 Category (Chapter) of Injury, poisoning, and certain other consequences of external causes (S00-T88). Among these encounters, 21.4% [95% CI, 21.3-21.4] met the criteria for the CHE 40 Risk and 28.5% [95% CI, 28.4-28.5] met criteria for the CHE 10 Risk threshold.

In **eFigure 12**, we use the data shown in eTable 8 to illustrate CHE 40 risk by disease category and disease prevalence (%).

© 2022 Scott et al. *JAMA Health Forum.*

eTable 7. ICD-10 CM Categories (Chapters), Code Range, and Titles

Chapter #	ICD-10 Range	ICD-10 CM Category Title	Short Title (For Charts)
**1**	A00-B99	Certain infectious and parasitic diseases	Infectious diseases
**2**	C00-D49	Neoplasms	Neoplasms
**3**	D50-D89	Diseases of the blood and blood-forming organs and certain disorders involving the immune mechanism	Hematologic and immunologic conditions
**4**	E00-E89	Endocrine, nutritional and metabolic diseases	Endocrine, nutritional and metabolic diseases
**5**	F01-F99	Mental, Behavioral and Neurodevelopmental disorders	Mental, behavioral and neurodevelopmental disorders
**6**	G00-G99	Diseases of the nervous system	Nervous system diseases
**7**	H00-H59	Diseases of the eye and adnexa	Diseases of the eye and adnexa
**8**	H60-H95	Diseases of the ear and mastoid process	Diseases of the ear and mastoid process
**9**	I00-I99	Diseases of the circulatory system	Circulatory diseases
**10**	J00-J99	Diseases of the respiratory system	Respiratory diseases
**11**	K00-K95	Diseases of the digestive system	Digestive diseases
**12**	L00-L99	Diseases of the skin and subcutaneous tissue	Skin diseases
**13**	M00-M99	Diseases of the musculoskeletal system and connective tissue	Musculoskeletal diseases
**14**	N00-N99	Diseases of the genitourinary system	Genitourinary diseases
**15**	O00-O9A	Pregnancy, childbirth and the puerperium	Pregnancy, childbirth and the puerperium
**16**	P00-P96	Certain conditions originating in the perinatal period	Perinatal conditions
**17**	Q00-Q99	Congenital malformations, deformations and chromosomal abnormalities	Congenital & chromosomal anomalies
**18**	R00-R99	Symptoms, signs and abnormal clinical and laboratory findings, not elsewhere classified	Symptoms, signs, and abnormal findings not otherwise classified
**19**	S00-T88	Injury, poisoning and certain other consequences of external causes	Injury, poisoning, & other external causes
**20**	V00-Y99	External causes of morbidity	External causes of morbidity
**21**	Z00-Z99	Factors influencing health status and contact with health services	Health status and health services influencers

Note: This is based on the 2017 CMS ICD-10-CM. More recent versions of the ICD-10 have an additional category (U00-U85: Codes for special purposes), but none of the observations mapped to these codes as this exploratory analysis included only 2017 data.

© 2022 Scott et al. *JAMA Health Forum.*

eTable 8. CHE Risk by Disease Category, Ranked by Disease Prevalence in Uninsured Treat-and-Release Sample

#	ICD-10 Chapter Title	ICD10 Range	Percent of Sample	CHE 40 Risk	CHE 40 95% C.I.	CHE 10 Risk	CHE 10 95% CI
**19**	Injury, poisoning and certain other consequences of external causes	S00-T88	20.2	21.4	(21.3-21.4)	28.5	(28.4-28.5)
**18**	Symptoms, signs and abnormal clinical and laboratory findings, not elsewhere classified	R00-R99	19.8	27.5	(27.5-27.6)	41.6	(41.5-41.6)
**10**	Diseases of the respiratory system	J00-J99	10.5	18.8	(18.8-18.9)	23.3	(23.3-23.4)
**13**	Diseases of the musculoskeletal system and connective tissue	M00-M99	8.9	19.2	(19.1-19.2)	24.2	(24.1-24.2)
**11**	Diseases of the digestive system	K00-K95	7.6	24.5	(24.4-24.6)	32.2	(32.0-32.3)
**14**	Diseases of the genitourinary system	N00-N99	6.8	28.1	(28.0-28.1)	42.6	(42.4-42.7)
**5**	Mental, Behavioral and Neurodevelopmental disorders	F01-F99	5.3	21.9	(21.8-22.0)	32.1	(31.9-32.2)
**12**	Diseases of the skin and subcutaneous tissue	L00-L99	5.0	17.1	(17.0-17.1)	18.4	(18.3-18.5)
**1**	Certain infectious and parasitic diseases	A00-B99	2.5	18.4	(18.3-18.5)	21.7	(21.5-21.9)
**21**	Factors influencing health status and contact with health services	Z00-Z99	2.4	14.7	(14.6-14.8)	13.9	(13.8-14.0)
**6**	Diseases of the nervous system	G00-G99	2.2	24.4	(24.3-24.5)	34.8	(34.6-35.0)
**15**	Pregnancy, childbirth and the puerperium	O00-O9A	2.1	25.0	(24.9-25.1)	38.8	(38.6-39.0)
**8**	Diseases of the ear and mastoid process	H60-H95	1.9	14.9	(14.8-14.9)	12.3	(12.2-12.4)
**9**	Diseases of the circulatory system	I00-I99	1.8	29.1	(28.9-29.2)	43.4	(43.2-43.7)
**4**	Endocrine, nutritional and metabolic diseases	E00-E89	1.4	28.6	(28.5-28.8)	45.2	(45.0-45.4)
**7**	Diseases of the eye and adnexa	H00-H59	1.2	15.6	(15.5-15.7)	13.3	(13.1-13.5)
**3**	Diseases of the blood and blood-forming organs and certain disorders involving the immune mechanism	D50-D89	0.3	33.7	(33.3-34.1)	52.7	(52.1-53.3)
**2**	Neoplasms	C00-D49	0.2	32.8	(32.3-33.3)	49.9	(49.1-50.7)
**16**	Certain conditions originating in the perinatal period	P00-P96	0.1	13.9	(13.5-14.2)	11.2	(10.6-11.8)
**17**	Congenital malformations, deformations and chromosomal abnormalities	Q00-Q99	<0.1	27.7	(26.3-29.2)	39.7	(37.3-42.1)
**20**	External causes of morbidity	V00-Y99	<0.1	24.3	(23.0-35.7)	33.7	(31.5-36.0)

© 2022 Scott et al. *JAMA Health Forum.*

eFigure 12. CHE Risk Among Uninsured ED Treat-and-Release Encounters by Disease Category and Disease Prevalence in Sample, 2017

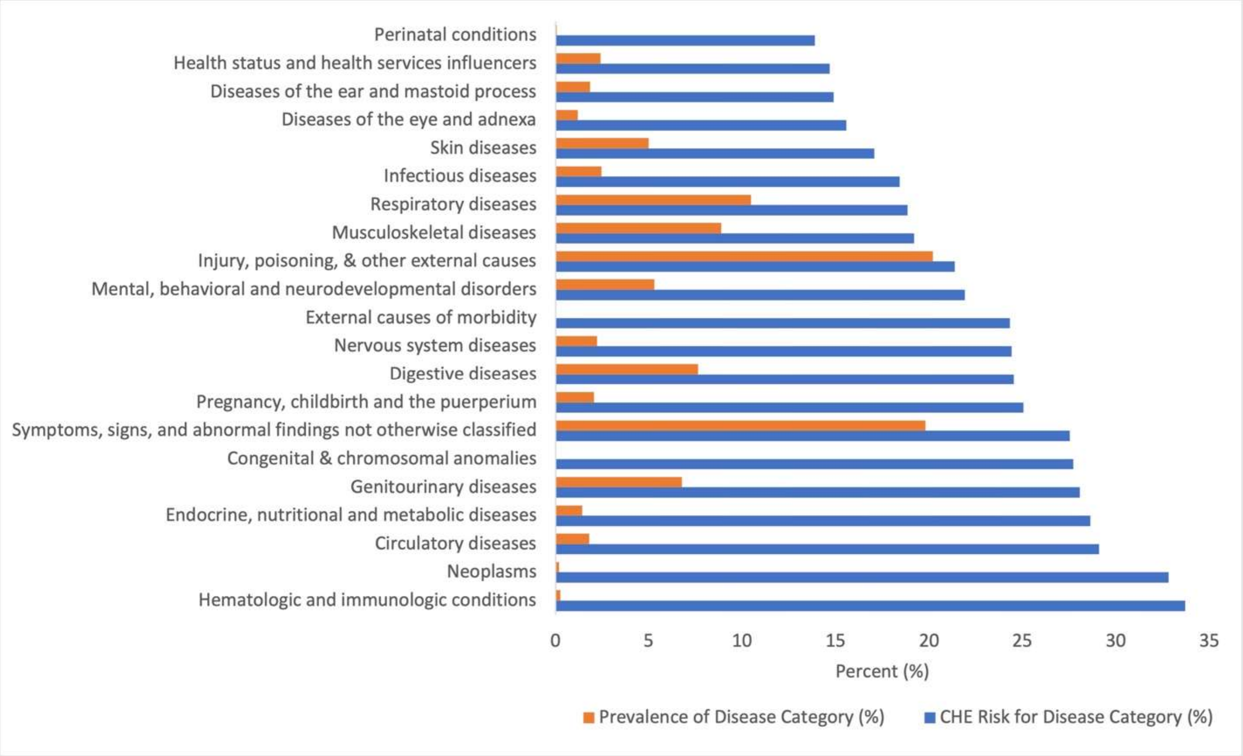

8. Additional Analysis of CHE Results

In **eTable 9**, we provide the unadjusted results for CHE risk for each covariate in this encounter-level study. The results come from a series of survey weighted linear regression models with CHE risk as the dependent variable and one covariate of interest (e.g., sex, age groups, income quartile, etc.) as the independent variable. After computing each regression model, the margins function was used to provide CHE risk (as opposed to the coefficient from the model output). A corresponding 95% confidence interval (CI) and p-value was provided. A p-value <0.05 suggests that there was a statistical difference in the predicted CHE risk between a group of the covariate as compared to the reference group within that variable (i.e., CHE risk for female versus male). Given the extremely large sample size available to us, it is not surprising that even small differences that may not be substantively different were statistically significant.

As shown in the primary analysis, we also provide the same results for each of the separate survey-weighed linear regression models containing both the covariate of interest as well as year fixed effects to account for secular trends. As before, the margins command was used to provide the estimated CHE risk for each variable that accounts for year fixed effects. As an example, encounters from the highest income quartile (Q4) had a predicted CHE risk of 6.6% as compared to 22.9% among the lowest income quartile (p-value <0.001), holding year constant.

eTable 9. CHE Risk for Each Covariates, Unadjusted Margins Versus Margins with Year Fixed Effects

		**Unadjusted Margins** *(without Year-Fixed Effects)*			**Margins** *(with Year-Fixed Effects)*		
**Demographic**		**CHE Risk (%)**	**95% C.I.**	**P-Value**	**CHE Risk (%)**	**95% C.I.**	**P-Value**
**Overall CHE Risk**		18.0	[18.0-18.0]	n/a	-	-	-
**Sex**	*Male*	17.8	[17.8-17.8]	REF	17.8	[17.8-17.8]	REF
	Female	18.2	[18.2-18.2]	<0.001	18.2	[18.2-18.2]	<0.001
**Age Groups**	*<20 years*	14.2	[14.2-14.3]	REF	14.7	[14.7-14.7]	REF
	20-44	18.0	[18.0-18.0]	<0.001	18.0	[18.0-18.0]	<0.001
	45-64	20.5	[20.5-20.5]	<0.001	20.3	[20.3-20.3]	<0.001
	65+	20.2	[20.2-20.3]	<0.001	20.0	[20.0-20.0]	<0.001
**Income Quartile**	*Highest (Q4)*	6.4	[6.4-6.4]	REF	6.6	[6.6-6.6]	REF
	Third (Q3)	12.9	[12.9-12.9]	<0.001	13.0	[13.0-13.0]	<0.001
	Second (Q2)	17.6	[17.6-17.6]	<0.001	17.7	[17.7-17.7]	<0.001
	Lowest (Q1)	23.1	[23.1-23.1]	<0.001	22.9	[22.9-22.9]	<0.001
**Rurality**	*Urban*	17.7	[17.7-17.7]	REF	17.6	[17.6-17.6]	REF
	Suburban	18.3	[18.3-18.3]	<0.001	18.3	[18.3-18.3]	<0.001
	Rural	18.2	[18.2-18.2]	<0.001	18.2	[18.2-18.2]	<0.001
**Hospital Region**	*Northeast*	14.7	[14.7-14.7]	REF	15.1	[15.1-15.1]	REF
	Midwest	17.0	[17.0-17.0]	<0.001	17.2	[17.1-17.2]	<0.001
	South	19.2	[19.2-19.2]	<0.001	19.1	[19.1-19.1]	<0.001
	West	19.2	[19.2-19.2]	<0.001	18.5	[18.5-18.5]	<0.001
**Teaching Status**	*Teaching Hospital (Metro)*	19.1	[19.1-19.1]	REF	18.6	[18.6-18.6]	REF
	Non-teaching Hospital (Metro)	17.5	[17.4-17.5]	<0.001	17.9	[17.9-17.9]	<0.001
	Rural	17.3	[17.3-17.3]	<0.001	17.5	[17.5-17.5]	<0.001
**Year**	*2006*	13.6	[13.6-13.6]	REF	-	-	-
	2007	13.8	[13.8-13.8]	<0.001	-	-	-
	2008	14.8	[14.8-14.8]	<0.001	-	-	-
	2009	15.1	[15.1-15.2]	<0.001	-	-	-
	2010	15.2	[15.2-15.2]	<0.001	-	-	-
	2011	17.8	[17.8-17.8]	<0.001	-	-	-
	2012	19.2	[19.2-19.2]	<0.001	-	-	-
	2013	21.0	[21.0-21.0]	<0.001	-	-	-
	2014	21.9	[21.9-21.9]	<0.001	-	-	-
	2015	20.4	[20.4-20.4]	<0.001	-	-	-
	2016	21.6	[21.6-21.6]	<0.001	-	-	-
	2017	22.6	[22.6-22.7]	<0.001	-	-	-

© 2022 Scott et al. *JAMA Health Forum.*

9. References

1. Xu K, Evans DB, Carrin G, Aguilar-Rivera AM, Musgrove P, Evans T. Protecting Households From Catastrophic Health Spending. *Health Affairs*. 2007;26(4):972-983. doi:10.1377/hlthaff.26.4.972

2. Xu K, Evans DB, Kawabata K, Zeramdini R, Klavus J, Murray CJ. Household catastrophic health expenditure: a multicountry analysis. *The Lancet*. 2003;362(9378):111-117. doi:10.1016/S0140-6736(03)13861-5

3. World Health Organization. *Designing Health Financing Systems to Reduce Catastrophic Health Expenditure*.; 2005. https://www.who.int/health_financing/pb_2.pdf

4. Scott JW, Raykar NP, Rose JA, et al. Cured into Destitution: Catastrophic Health Expenditure Risk Among Uninsured Trauma Patients in the United States. *Annals of Surgery*. 2018;267(6):1093-1099. doi:10.1097/SLA.0000000000002254

5. Khera R, Hong JC, Saxena A, et al. Burden of Catastrophic Health Expenditures for Acute Myocardial Infarction and Stroke Among Uninsured in the United States. *Circulation*. 2018;137(4):408-410. doi:10.1161/CIRCULATIONAHA.117.030128

6. Agency for Healthcare Research and Quality (AHRQ). THE HCUP NATIONWIDE EMERGENCY DEPARTMENT SAMPLE (NEDS), 2017. Accessed October 2, 2020. https://www.hcup-us.ahrq.gov/db/nation/neds/NEDS_Introduction_2017.jsp

7. World Bank. Gini index (World Bank estimate) - United States | Data. Accessed August 10, 2021. https://data.worldbank.org/indicator/SI.POV.GINI?locations=US

8. Salem ABZ, Mount TD. A Convenient Descriptive Model of Income Distribution: The Gamma Density. *Econometrica*. 1974;42(6):1115-1127. doi:10.2307/1914221

9. Shrime MG, Dare A, Alkire BC, Meara JG. A global country-level comparison of the financial burden of surgery. *BJS*. 2016;103(11):1453-1461. doi:10.1002/bjs.10249

10. Farooq A, Merath K, Hyer JM, et al. Financial toxicity risk among adult patients undergoing cancer surgery in the United States: An analysis of the National Inpatient Sample. *J Surg Oncol*. 2019;120(3):397-406. doi:10.1002/jso.25605

11. Scott JW, Sommers BD, Tsai TC, Scott KW, Schwartz AL, Song Z. Dependent Coverage Provision Led To Uneven Insurance Gains And Unchanged Mortality Rates In Young Adult Trauma Patients. *Health Aff*. 2015;34(1):125-133. doi:10.1377/hlthaff.2014.0880

12. Ranson MK. Reduction of catastrophic health care expenditures by a community-based health insurance scheme in Gujarat, India: current experiences and challenges. *Bulletin of the World Health Organization*. 2002;80:613-621.

13. Devadasan N, Criel B, Van Damme W, Ranson K, Van der Stuyft P. Indian community health insurance schemes provide partial protection against catastrophic health expenditure. *BMC health services research*. 2007;7(1):1-11.

14. Shrime MG, Dare AJ, Alkire BC, O'Neill K, Meara JG. Catastrophic expenditure to pay for surgery worldwide: a modelling study. *Lancet Glob Health*. 2015;3 Suppl 2:S38-44. doi:10.1016/S2214-109X(15)70085-9

15. Centers for Medicare & Medicaid Services. 2017 ICD-10-CM and GEMs | CMS. 2017 ICD-10-CM and GEMs. Accessed September 1, 2021. https://www.cms.gov/Medicare/Coding/ICD10/2017-ICD-10-CM-and-GEMs

© 2022 Scott et al. *JAMA Health Forum.*
